# Polarized branched Actin modulates cortical mechanics to produce unequal-size daughters during asymmetric division

**DOI:** 10.1038/s41556-022-01058-9

**Published:** 2023-02-06

**Authors:** Alicia Daeden, Alexander Mietke, Emmanuel Derivery, Carole Seum, Frank Jülicher, Marcos Gonzalez-Gaitan

**Affiliations:** 1 Department of Biochemistry, Faculty of Sciences, University of Geneva, Geneva, Switzerland; 2 Department of Mathematics, Massachusetts Institute of Technology, Cambridge, MA USA; 3 Max Planck Institute for the Physics of Complex Systems, Dresden, Germany; 4 Max Planck Institute of Molecular Cell Biology and Genetics, Dresden, Germany; 5 Center for Systems Biology Dresden, Dresden, Germany; 6 MRC Laboratory of Molecular Biology, Cambridge, UK

**Keywords:** Actin, Cytokinesis, Biophysics

## Abstract

The control of cell shape during cytokinesis requires a precise regulation of mechanical properties of the cell cortex. Only few studies have addressed the mechanisms underlying the robust production of unequal-sized daughters during asymmetric cell division. Here we report that unequal daughter-cell sizes resulting from asymmetric sensory organ precursor divisions in *Drosophila* are controlled by the relative amount of cortical branched Actin between the two cell poles. We demonstrate this by mistargeting the machinery for branched Actin dynamics using nanobodies and optogenetics. We can thereby engineer the cell shape with temporal precision and thus the daughter-cell size at different stages of cytokinesis. Most strikingly, inverting cortical Actin asymmetry causes an inversion of daughter-cell sizes. Our findings uncover the physical mechanism by which the sensory organ precursor mother cell controls relative daughter-cell size: polarized cortical Actin modulates the cortical bending rigidity to set the cell surface curvature, stabilize the division and ultimately lead to unequal daughter-cell size.

## Abstract (web summary)

Daeden et al. report that branched Actin modulates size asymmetry in *Drosophila* sensory organ precursor cell division.

## Main

Many asymmetric cell divisions (ACDs) are characterized by a difference in size of the two daughter cells^1^. From a physical point of view, the generation of unequal daughter-cell size represents a mechanical challenge during cytokinesis. According to Laplace law, if two daughter cells are not identical in size, the smaller daughter would collapse into the bigger daughter during cytokinesis. Indeed, cells, dividing under a global contractile tension at the poles, are expected to exhibit dramatic shape instabilities^2,3^. As daughter cells are never exactly identical, cells must ensure to compensate for such instabilities, which can ultimately lead to cytokinetic failure^2^. Consequently, the precise control of the mechanical properties at the cortical poles is critical during ACD, when cells divide into unequal daughter-cell size. Identifying the mechanisms of daughter-cell size control is also crucial to understand cell function, because the ability to produce asymmetric daughters underlies cellular diversity and can influence proliferation rates^4^ and cell differentiation^4–6^, which are key for stem cell renewal^7–9^.

Previous reports have proposed a role of spindle positioning in determining size asymmetry^10–12^. However, recent data suggest that spindle-induced cleavage furrow positioning is not enough to explain cell-size asymmetry (reviewed in ref. ^13^) and cells without spindles can divide with normal daughter-cell size asymmetry^14,15^. Furthermore, this does not resolve how the cell overcomes the mechanical instabilities during cytokinesis mentioned above.

Instead, the actomyosin cortex at the cell poles has been proposed to play a key role in the generation of cell size asymmetry^5,14,15^. These previous studies have mainly focused on the role of contractile actomyosin tension in determining cortical mechanical properties, while the role of other mechanical properties, such as cortical stiffness or bending rigidity, is less well understood. For example, during asymmetric neuroblast division in *Caenorhabditis elegans* and *Drosophila*, cortical Myosin enrichment in the smaller pole has been proposed to produce unequal-sized daughters by creating an asymmetry of contractile tension^5,14,15^. However, during these divisions, it is unknown how shape instabilities, such as the collapse of the smaller cell pole, which exhibits a larger contractile tension, are prevented^2^. Thus, how cells control and stabilize their shape and relative daughter-cell size during ACD remains an open question. In this Article, we investigate how polar branched Actin at the cell cortex regulates cortical mechanics to robustly achieve asymmetric division into two unequal-sized daughter cells.

## Results

### Daughter-cell size asymmetry during SOP division

**Fig. 1:**
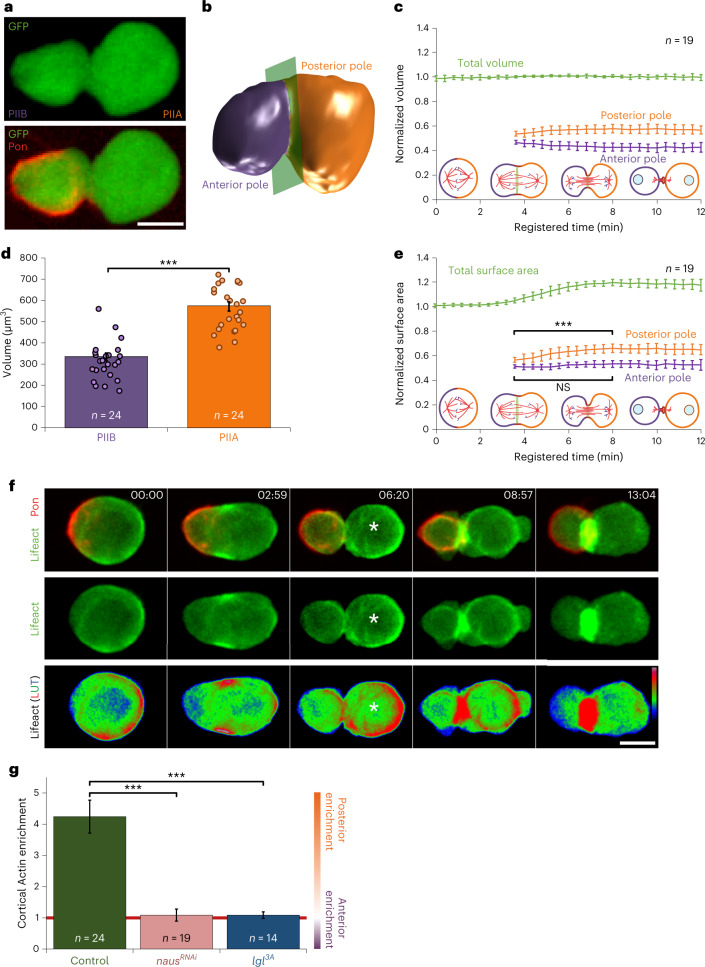
Daughter-cell size and cortical Actin asymmetry. **a**, Maximum projection of SOP expressing cytosolic GFP (green) and RFP-Pon (red; a marker of the anterior pole corresponding to the future PIIB cell) under the *Neuralized* promoter. **b**, Three-dimensional segmentation of a dividing SOP using the Squassh algorithm (Supplementary Note 1); anterior (violet) and posterior cell poles (orange) are indicated. Green plane, cleavage plane. **c**, Volume dynamics (total volume and volume of the poles) during mitosis (mean ± standard deviation). Cytokinesis stages are indicated. Volumes have been normalized by the average conserved volume of each cell, respectively (Supplementary Note 1). Poles are defined only after the onset of cytokinesis (at the beginning of anaphase B) by the position of the cleavage furrow. **d**, Daughter-cell volume. Mean ± s.e.m., *t*-test two-tailed, *P* < 0.001. **e**, Surface area dynamics (total and polar) during mitosis (mean ± standard deviation). Areas have been normalized by the corresponding area from a sphere of volume equals to the total average volume for each cell, respectively (Supplementary Note 1). Poles are defined only after the onset of cytokinesis (at the beginning of anaphase B) by the position of the cleavage furrow. Mann–Whitney rank sum test two-sided (*P* < 0.001 for posterior and not significant (NS) for anterior). **f**, Top: time-lapse spinning disk confocal maximum projections of SOP expressing Lifeact-mCherry (red) and GFP-Pon (green) under the *Neuralized* promoter. Middle: a posterior enrichment of cortical Actin (Lifeact) can be observed in late anaphase (see third timepoint, white asterisk). Bottom: look-up table (LUT): redder pixels correspond to high fluorescence signals. Note that Pon refers to the membrane targeting domain of Pon, not the full length. **g**, Cortical Actin enrichment in the posterior cortex in control, UAS-*nausicaa*^*RNAi*^ (*naus*^*RNAi*^) and UAS-*lgl*^*3A*^ (*lgl*^*3A*^) conditions (posterior-to-anterior ratio measured by ‘Averaged Linescans’ method; Supplementary Note 1). Red line: symmetrical daughter-cell sizes. Mean ± s.e.m.; control, *n* = 24; *naus*^*RNAi*^, *n* = 19; *lgl*^*3A*^, *n* = 14; Kruskal–Wallis one-way ANOVA on ranks, followed by a Dunn’s post hoc test, *P* < 0.001. Scale bars, 5 µm. For details on genotypes, in this and other figures in this report, see Supplementary Table 1. *n* indicates number of cells from 6, 6 and 6 pupae for **c**, **d** and **e**, respectively, and from 8, 8 and 5 pupae for control, *naus*^*RNAi*^ and *lgl*^*3A*^ measurements, respectively, in **f**. Source numerical data are available in source data. Source data

**Extended Data Fig. 1:**
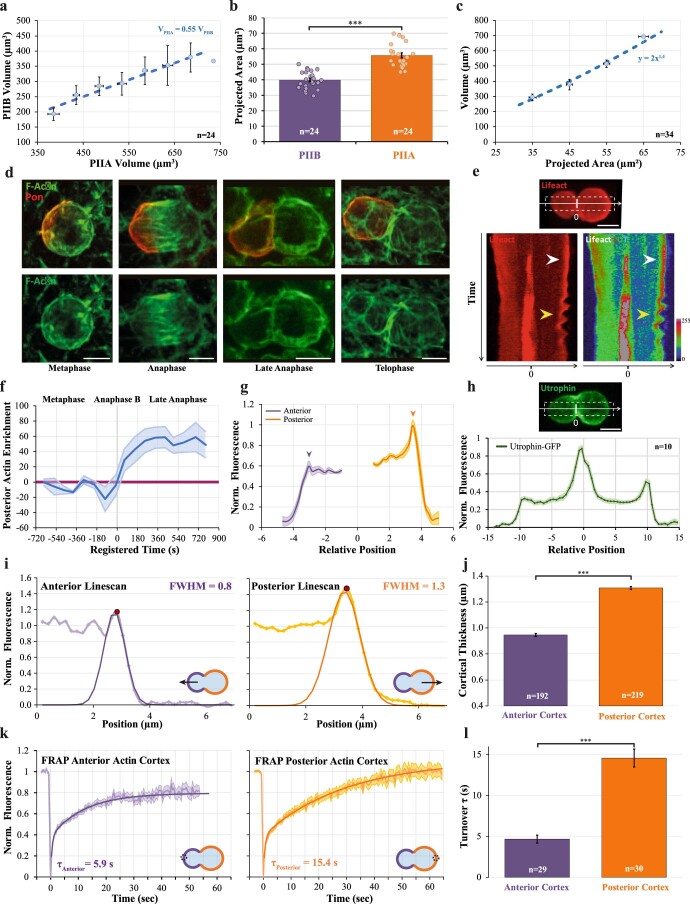
Daughter-cell size and cortical Actin asymmetry. **a)** PIIA *versus* PIIB daughter-cell volumes (Correlation factor: V_PIIA_ = 0.55V_PIIB_). **b)** Projected Area (PA) measurements for each daughter-cell (PA_PIIA_ / PA_PIIB_ = 1.4 ± 0.2; n = 24, T-Test two-tailed, P < 0.001). **c)** Correlation between volume and projected area of the two daughter-cells. Projected area measurement is a reliable proxy for daughter-cell volumes. Power law correlation (dashed line; R² = 0.98). **d)** Phalloidin staining in SOPs expressing mRFP-Pon revealing filamentous Actin (F-Actin). **e)** Kymographs of the Lifeact signal along cytokinesis. For representation purpose, intensity has been color-coded using the Rainbow lookup table (red: left and LUT: right). White arrowheads: posterior cortical Actin enrichment; Yellow arrowheads: blebbing phenomena occurring in late cytokinesis stages mostly in the posterior compartment (See Extended Data Fig. 9a, b). **f)** Dynamics of the cortical Actin posterior enrichment in SOPs expressing Lifeact-mCherry (n = 4; Average + /− StDev). Posterior Actin Enrichment = $\frac{{{{{\mathrm{Posterior}}}}_{{{{\mathrm{Cortical}}}}\,{{{\mathrm{Actin}}}}}-{{{\mathrm{Anterior}}}}_{{{{\mathrm{Cortical}}}}\,{{{\mathrm{Actin}}}}}}}{{{{{\mathrm{Anterior}}}}_{{{{\mathrm{Cortical}}}}\,{{{\mathrm{Actin}}}}}}} \times 100$ (see Supplementary Methods). **g)** Averaged signal intensities from linescans across SOPs in late anaphase expressing Lifeact mCherry (n = 8). Violet arrowhead = anterior cortical Actin; Orange arrowhead = posterior cortical Actin. **h)** Upper panel: SOP expressing UAS-Utrophin-GFP (a marker for F-Actin) driven by the *Neuralized* promoter. Lower panel: Signal intensity from linescan of the Utrophin signal. **i)** FWHM measurements from linescans over the anterior cortex (left) or posterior cortex (right) from SOP expressing Lifeact mCherry (see Supplementary Methods). **j)** FWHM values as a proxy of the cortical thickness between the anterior and the posterior cortex. Mann-Whitney Rank Sum Test two-sided, P < 0.001. **k)** Fluorescence recovery monitored over time after photobleaching (FRAP) of GFP-Actin at the cell cortex (Left: FRAP at anterior cortex; Right: FRAP at the posterior cortex; Mean + /- SEM). Curves were fitted to a double exponential model (see **Methods**, violet and oranges lines, respectively). **l)** Average turnovers of GFP-Actin from FRAP experiments on the anterior and posterior cortex (average of the two *τ* from the double exponential fit: ($\frac{{\tau 1 + \tau 2}}{2}$), for the anterior pole *τ*_1_ = 0.3 ± 0.08; *τ*_2_ = 9.01 ± 0.9 and for the posterior pole *τ*_1_ = 0.8 ± 0.32; *τ*_2_ = 28.31 ± 2.2). Mann-Whitney Rank Sum Test two-sided, P < 0.001. All data are presented as Mean ± SEM unless stated otherwise. For details on genotypes, in this and other figures in this report, see Supplementary Table 1. *n* = number of cells from 6, 6, 6, 2, 4, 4, 9, 10 pupae for **a**, **b**, **c**, **f**, **g**, **h**, **j**, **l** respectively. Source numerical data are available in source data. Source data

Sensory organ precursor (SOP) cells divide into an anterior PIIB and a posterior PIIA daughter cell. To study the sizes of PIIA and PIIB (Fig. 1a), we analysed the three-dimensional (3D) geometry of dividing SOPs from imaging data (Fig. 1b and Supplementary Note 1). During cytokinesis, SOP volume remains constant, and, at the end of mitosis, PIIA has nearly twice the volume of PIIB (Fig. 1c,d and Extended Data Fig. 1a–c). The total surface area increases by 20%, as expected for a sphere when it is split into two with volume conservation (Fig. 1e and Supplementary Information II.I). Using the cleavage furrow as landmark, we found that the increase of total surface area is mainly due to an increase in posterior pole surface (Fig. 1e). To identify the origin of this surface area asymmetry and corresponding cell size asymmetry, we studied the cortical cytoskeleton at the poles.

### Cortical Actin asymmetry during asymmetric mitosis

**Extended Data Fig. 2:**
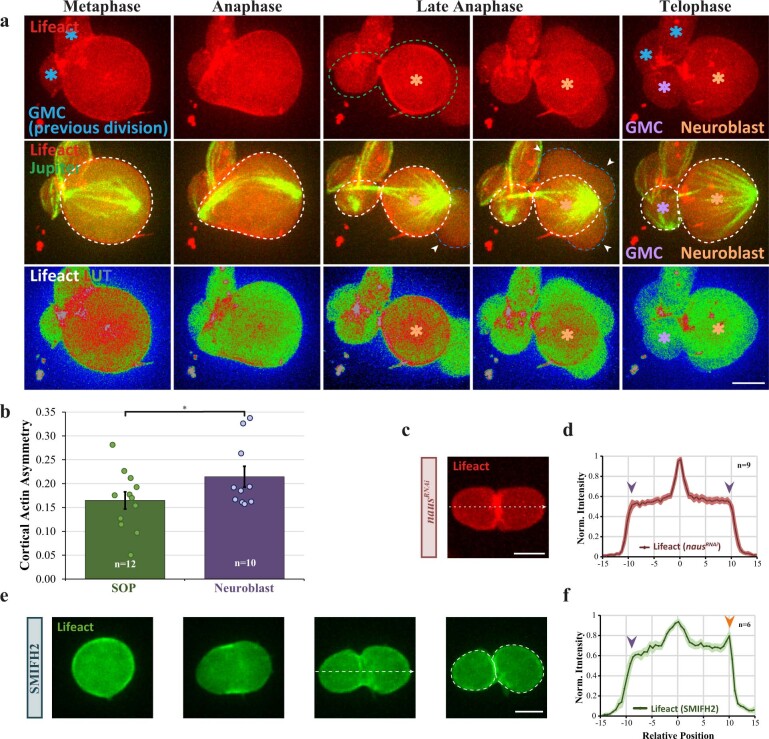
Cortical Actin asymmetry: Neuroblasts and importance of branched Actin. **a)** Time-lapse spinning disk confocal imaging of representative dividing Neuroblast (from *Drosophila* brain primary culture) expressing Jupiter-GFP and Lifeact-mCherry (in red, upper panel and LUT, lower panel). Blue asterisks: GMC (ganglion mother cell) daughter-cells from precedent divisions. The neuroblast division (dashed white line) gives rise to a newly formed GMC (violet asterisk, smaller daughter-cell; basal side in whole brain) and a newly formed neural stem cell (orange asterisk, apical pole, bigger daughter-cell). Note the enrichment of Actin in the apical pole (bigger pole, future neuroblast, orange asterisk) in the third timepoint. White arrowheads and dashed blue lines highlight blebs in the apical pole in the third and fourth timepoints (middle panel). **b)** Cortical Actin asymmetry in the posterior cortex in SOPs *versus* Neuroblasts. Actin asymmetry=($\frac{{{{{\mathrm{I}}}}_{{{{\mathrm{Posterior}}}}\,{{{\mathrm{Cortical}}}}\,{{{\mathrm{Actin}}}}} - {{{\mathrm{I}}}}_{{{{\mathrm{Anterior}}}}\,{{{\mathrm{Cortical}}}}\,{{{\mathrm{Actin}}}}}}}{{{{{\mathrm{I}}}}_{{{{\mathrm{Anterior}}}}\,{{{\mathrm{Cortical}}}}\,{{{\mathrm{Actin}}}}} + {{{\mathrm{I}}}}_{{{{\mathrm{Posterior}}}}\,{{{\mathrm{Cortical}}}}\,{{{\mathrm{Actin}}}}}}}$) measured by the ‘Linescan’ method - See Supplementary Methods). T-Test one-tailed (P < 0.05). **c**,**d)** Maximum projection (**c**) and corresponding linescans of the Lifeact-mCherry signal (**d**) of SOPs expressing *nausicaa*^*RNAi*^ (*naus*^*RNAi*^; pink) together with Lifeact-mCherry under the *Neuralized* promoter. Arrowheads, cortical Actin. Note the loss of the cortical Actin asymmetry in *naus*^*RNAi*^ conditions. **e**,**f)** Time-lapse spinning disk confocal imaging of a SOP expressing Lifeact-mCherry under the *Neuralized* promoter and incubated with the SMIFH2 Formin inhibitor drug (**e**) and corresponding linescan of the Lifeact-GFP signal (**f**). Note that upon addition of the SMIFH2 drug, both daughter size and Actin asymmetries are still visible. All data are presented as mean values +/− SEM. *n* = number of cells from 4 pupae for SOPs and 6 third instar larvae for Neuroblasts in **b** and 4, 3 pupae in **d**, **f** respectively. Scale bar 5μm. Source numerical data are available in source data. Source data

As in other mitotic systems^16,17^, Actin is recruited to the SOP cortex at metaphase onset (Extended Data Fig. 1d) during the mitotic rounding process^18–20^. In contrast, in late anaphase, the posterior Actin cortex is enriched by fourfold (Fig. 1f,g, Supplementary Video 1 and Extended Data Fig. 1d–h) and is 40% thicker compared with the anterior cortex (Extended Data Fig. 1i,j). In accordance with this, Actin dynamics in the posterior cortex is slower, as revealed by fluorescence recovery after photobleaching (FRAP) using green fluorescent protein (GFP)-Actin (Extended Data Fig. 1k,l). Similar to the SOP, *Drosophila* larval neuroblasts also display asymmetric cortical Actin enrichment, with higher levels in the apical pole, which gives rise to the neural stem cell, the larger daughter (Extended Data Fig. 2a,b).

**Extended Data Fig. 3:**
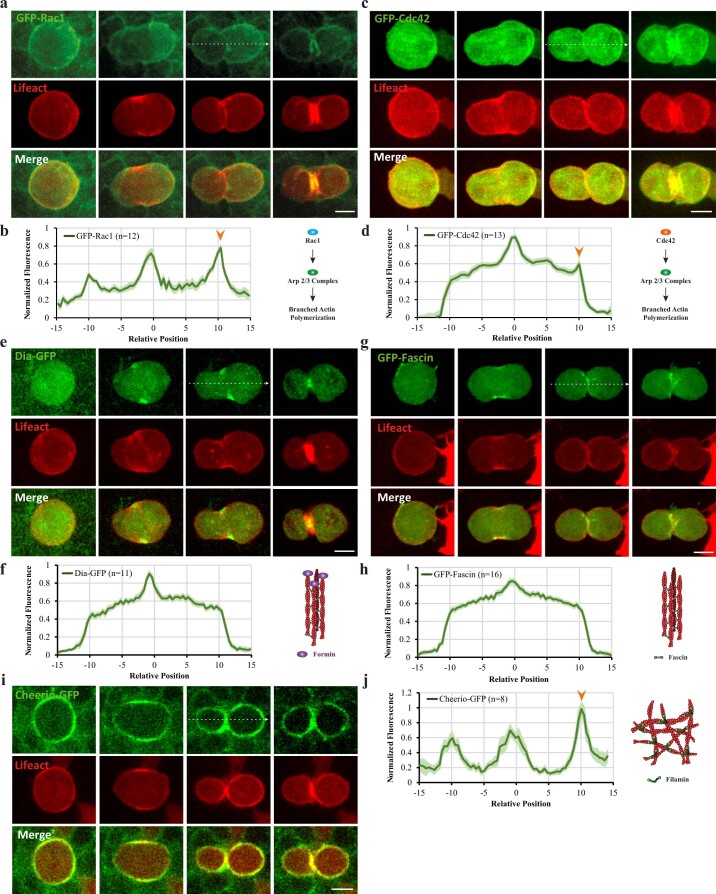
A branched and crosslinked posterior cortical Actin. **a-j)** Time-lapse spinning disk confocal imaging of a SOP expressing endogenous GFP-Rac1 (Ras-related GTPases regulating branched F-Actin growth) (**a**), UAS GFP-Cdc42 (Ras-related GTPases regulating branched F-Actin growth) (**c**), Dia-GFP (Diaphanous-related Formin, Formins Actin nucleators regulating linear long F-Actin (**e**), UAS Fascin-GFP (**g**) and Cheerio-GFP (*Drosophila* ortholog of mammalian Filamin, Actin Crosslinker; one z-plane) (**i**) driven by the *Neuralized* promoter. Averaged signal intensities in **b**, **d**, **f**, **h**, **j** from linescans across SOPs in late anaphase to visualize the fluorescence signal observed in **a**, **c**, **e**, **g**, **i**. Note the accumulation of Rac (**b**), Cdc42 (**d**) and Filamin (**j**) in the posterior cortex (arrowheads) and the absence of cortical accumulation of Formins (**f**) and Fascin (**h**) at the cell poles. Note that Rac1 and Cdc42 tagged proteins might reflect both an active and an inactive pool. It is also worth noting that, in other organisms, Rac has been reported to bind to some Formins (such as FHOD in Mouse fibroblasts^94^), although the functional relevance of this interaction remains unclear^95,96^. Also, Cdc42 has been reported to be upstream Formins during planar cell polarity in the *Drosophila* eye^97^. Scale bars 5μm. All data are presented as Mean ± SEM. *n* = number of cells from 4, 4, 3, 5, 3 pupae for **b**, **d**, **f**, **h**, **j** respectively. Source numerical data are available in source data. Source data

**Extended Data Fig. 4:**
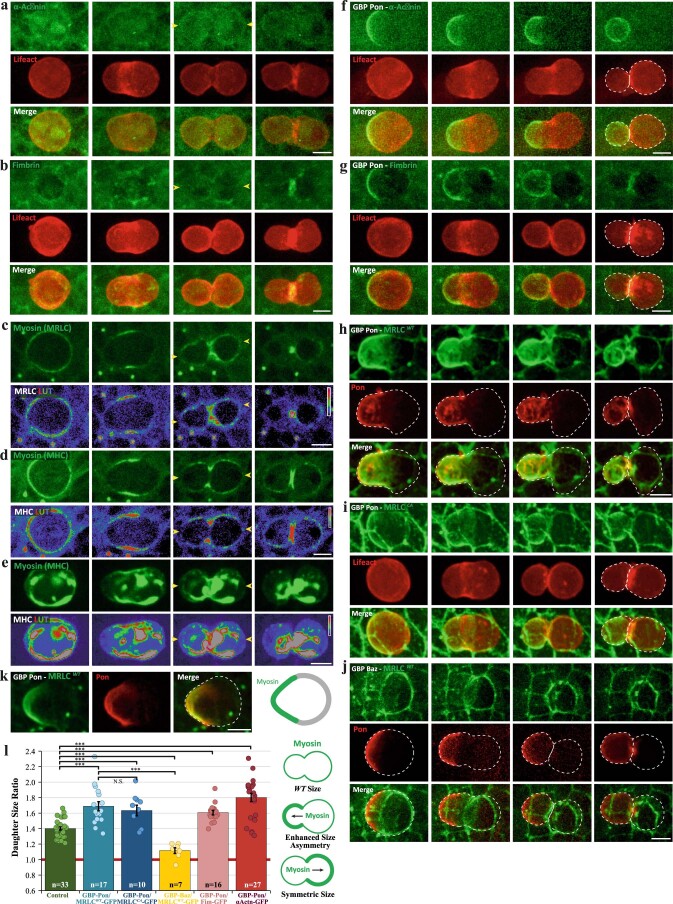
Polar targeting of Myosin and Actin bundling crosslinkers decreases daughter-cell size. **a**, **b**) Time-lapse spinning disk confocal images showing expression of Lifeact-mCherry (**a**, **b**; red) under the under the *Neutralized* promoter in SOPs together with expression of endogenous α-Actinin-GFP (**a**; green) or endogenous Fimbrin-GFP (**b**; green) in the whole epithelium. Note the absence of posterior cortical enrichment or asymmetry for either α-Actinin-GFP and Fimbrin-GFP (yellow arrowheads, third timepoint). **c**,**d**) Time-lapse spinning disk confocal images showing expression under their own promoter in the whole epithelium of Myosin Regulatory Light Chain (MRLC; green; **c**) or Myosin Heavy Chain (MHC; red; **d**) fused to GFP. One z plane. **e**) Overexpression of MHC-GFP under the *Neutralized* promoter in SOPs. Maximal projection. Upon MHC overexpression, aggregates of myosin motors are observed. We could not detect any asymmetry in the cortical Myosin between the two cell poles (third timepoints in (**c-e**); yellow arrowheads). Rainbow LUT (lower panel of **c-e**). **f**,**g**) Time-lapse spinning disk confocal images showing expression of Lifeact-mCherry (red) together with the nanobody GBP-Pon (untagged) under the under the *Neutralized* promoter in SOPs as well as expression of endogenous α-Actinin-GFP (**f**; green) or endogenous Fimbrin-GFP (**g**; green) in the whole epithelium. **h-j**) Time-lapse spinning disk confocal images showing expression of RFP-Pon (**h**, **j**; red), Lifeact-mCherry (**i**; red), GBP-Pon, (**h**, **i**) or GBP-Baz (**j**) under the under the *Neutralized* promoter in SOPs as well as expression under their own promoter, in the whole epithelium of MRLC^WT^-GFP (**h**, **j**; green) or constitutively active MRLC^CA^ (**i**; green), **k**) Metaphase SOP expressing MRLC-GFP under its own promoter in the whole epithelium (green) as well as UAS-RFP-Pon and UAS-GBP-Pon under the *Neutralized* promoter in SOPs. We observed a higher curvature in the anterior pole where Myosin is targeted (**k**), leading ultimately to a smaller anterior daughter-cell (**h**, **i**, **l**). **l**) Daughter-cell projected area ratios showing an enhancement of the daughter-cell size asymmetry when Myosin is targeted to the anterior cortex or a more symmetrical daughter sizes when Myosin is targeted to the posterior cortex (Kruskal-Wallis non-parametric one-way ANOVA followed by Holm-Sidak test, P < 0.001). Data are presented as Mean ± SEM. *n* = number of cells in **l** from 10, 5, 3, 5, 6, 9 pupae from left to right respectively. Scale Bar 5μm. Source numerical data are available in source data. Source data

Formins and the Arp2/3 complex drive the polymerization of linear and branched filamentous Actin (F-Actin), respectively^21–23^. In SOPs, we show that asymmetric cortical F-Actin probably arises from an asymmetric Arp2/3 activation. Indeed, when Nausicaa, a protein known to regulate branching nucleation and density in *Drosophila*^24^, is downregulated, Actin asymmetry is abolished (Fig. 1g and Extended Data Fig. 2c,d). Conversely, when Formins are inhibited by the SMIFH2 drug^25–27^, cortical Actin asymmetry is not affected (Extended Data Fig. 2e,f). Furthermore, both Rac and Cdc42, upstream regulators of Arp2/3 (ref. ^28^), are enriched in the posterior cortex (Extended Data Fig. 3a–d), but not Diaphanous, which is the main Formin known to nucleate Actin at the cell cortex and to be involved during cytokinesis^21,29–31^ (Extended Data Fig. 3e,f). Additionally, only Filamin (a large, flexible crosslinker arranging Actin into a meshwork)^32,33^ is found enriched at the cortical poles, but not Fascin, α-Actinin or Fimbrin, all of which organize F-Actin into bundles^34–36^ (Extended Data Figs. 3g–j and 4a,b). Therefore, Actin asymmetry seems to be dominated by branched Actin in SOPs.

In other ACDs, enrichment of cortical Myosin has been shown to correlate with the smaller daughter cell^5,14,15^. We then set up to study and manipulate contractility factors (such as Myosin and bundler proteins) in SOPs (Extended Data Fig. 4a–l). For instance, Myosin-II is found enriched at the cell cortex, but no asymmetry could be detected (Extended Data Fig. 4c–e). Therefore, asymmetric cell size in SOPs might arise from an alternative mechanism, where the asymmetric accumulation of branched cortical Actin, instead of Myosin, determines relative daughter-cell sizes. To investigate this, we next studied the functional relationship between cortical Actin asymmetry and unequal daughter-cell size.

### Actin asymmetry coupled with PAR complex and daughter size

Cell size asymmetry during ACD can be controlled through polarity cues organized by the PAR complex^37^. We thus studied cortical Actin in SOPs expressing a phospho-defective *lgl* mutant version (*lgl*^*3A*^) known to impair PAR complex activity^38^.

**Fig. 2:**
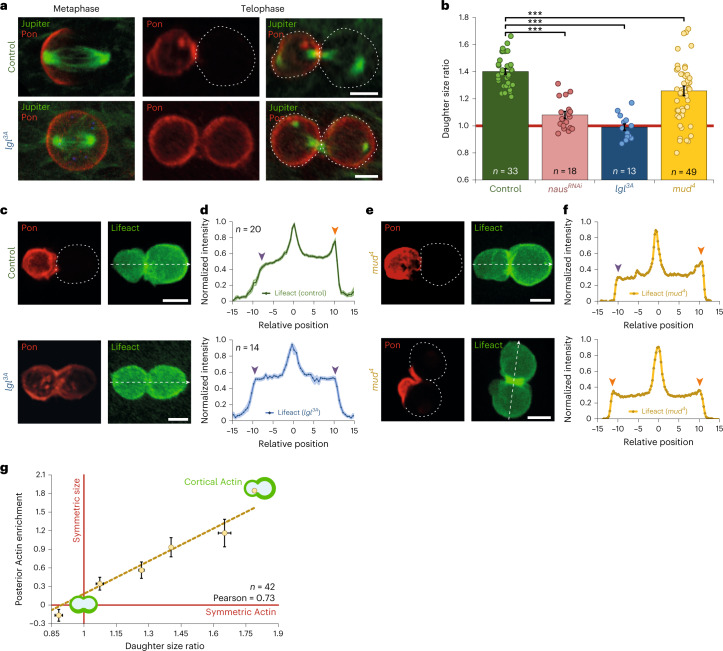
Cortical Actin asymmetry correlates with daughter-cell size asymmetry. **a**, Maximum projection of SOPs expressing Jupiter-mCherry (green) and GFP-Pon (red) in control (top) and in cells expressing UAS-*lgl*^*3A*^ under the *Neuralized* promoter (bottom). Dashed line, cell contour based on low level GFP/RFP signals. **b**, Projected area ratio of daughter cells ($\frac{{{{{\mathrm{Area}}}}_{{{{\mathrm{Posterior}}}}\,{{{\mathrm{PIIA}}}}}}}{{{{{\mathrm{Area}}}}_{{{{\mathrm{Anterior}}}}\,{{{\mathrm{PIIB}}}}}}}$); Kruskal–Wallis non-parametric one-way ANOVA followed by Dunn’s post hoc test (*P* < 0.001). Projected area corresponds to the surface of the *z*-projected SOP as a proxy of the volume (Extended Data Fig. 1c). Note the broad range of ratios in *mud*^*4*^ mutants. **c**, Maximum projection of SOP cells expressing Lifeact-mCherry and GFP-Pon in control (top) and in cells expressing UAS-*lgl*^*3A*^ under the *Neuralized* promoter (bottom). These results were confirmed by using mutants on factors upstream the PAR complex: the PCP gene *dishevelled*^66^ and *Gβ13F*^67,68^ (Extended Data Fig. 5a-e). **d**, Signal intensity along linescans (20-pixel width linescan centred around the white dashed lines) of Lifeact-mCherry (Supplementary Note 1). Arrowheads, cortical Actin. **e**, Maximum projection of SOP cells expressing Lifeact-mCherry and GFP-Pon under the *Neuralized* promoter in *mud*^*4*^ mutant conditions. Two examples of randomized orientations of the mitotic plane with respect to the PAR polarity complex axis. Note that we never observed formation of ‘polar lobes’ in mutant SOPs, unlike what was reported for *mud*^*4*^ mutant neuroblasts^14^. **f**, Signal intensity along linescans of Lifeact-mCherry in *mud*^*4*^ mutant conditions (**e**). **g**, Posterior cortical Actin enrichment ($\frac{{{{I}}_{{{{\mathrm{Posterior}}}}\,{{{\mathrm{cortical}}}}\,{{{\mathrm{Actin}}}}}-{{I}}_{{{{\mathrm{Anterior}}}}\,{{{\mathrm{cortical}}}}\,{{{\mathrm{Actin}}}}}}}{{{{I}}_{{{{\mathrm{Anterior}}}}\,{{{\mathrm{cortical}}}}\,{{{\mathrm{Actin}}}}} + {{I}}_{{{{\mathrm{Posterior}}}}\,{{{\mathrm{cortical}}}}\,{{{\mathrm{Actin}}}}}}}$) measured by the ‘Linescan’ method (Supplementary Note 1) versus daughter-cell size ratio ($\frac{{{{{\mathrm{Area}}}}_{{{{\mathrm{Posterior}}}}}}}{{{{{\mathrm{Area}}}}_{{{{\mathrm{Anterior}}}}}}}$) in *mud*^4^ mutant. Dashed line, linear fit. Here posterior pole is defined as the pole with the lowest GFP-Pon signal. *n* indicates number of cells from 10, 8, 5 and 16 pupae in **b** for control, *naus*^*RNAi*^, *lgl*^*3A*^ and *mud*^*4*^ measurements respectively, from 8 and 5 pupae for **d** and 16 pupae for **g**. All data are presented as mean ± s.e.m. Scale bars, 5 µm. Source numerical data are available in source data. Source data

**Extended Data Fig. 5:**
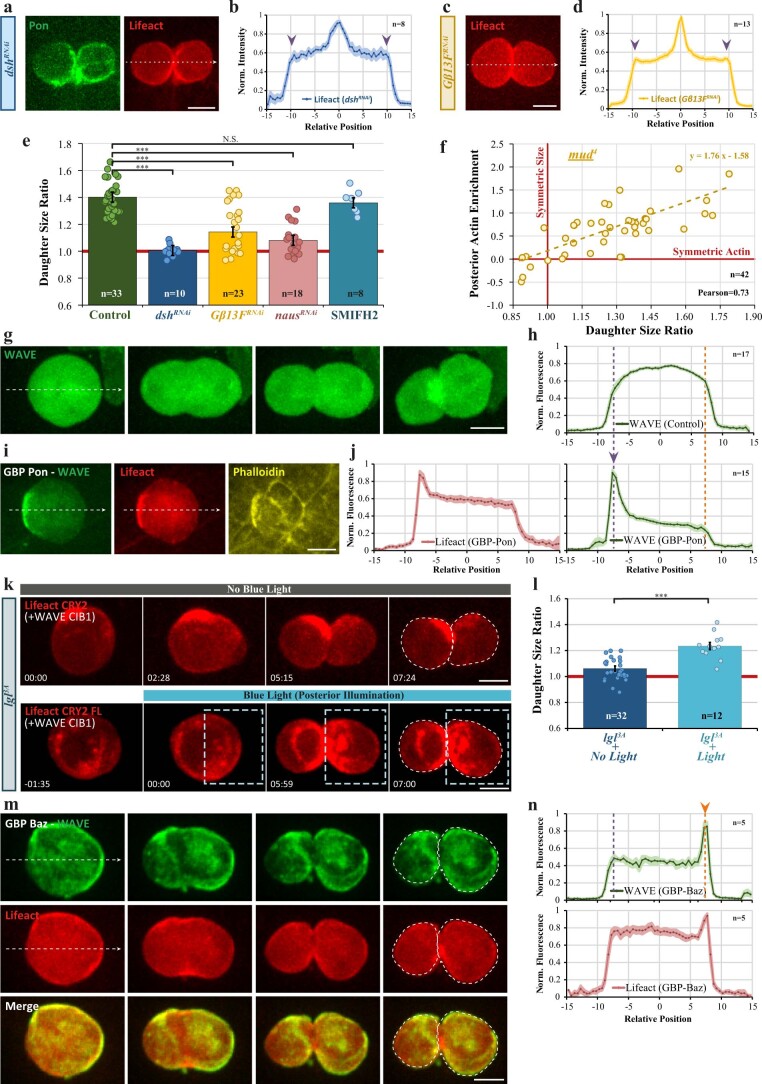
Other polarity cues, optogenetics and nanobody mistargeting of WAVE. **a-d)** Maximum projection (**a**, **c**) and corresponding linescans of the Lifeact-mCherry signal (**b**, **d**) of SOPs expressing GFP-Pon and *dishevelled*^*RNAi*^ (**a**: *dsh*^*RNAi*^; blue) or the heterotrimeric protein *Gβ13F*^*RNAi*^ (**c**: *Gβ13F*^*RNAi*^; yellow) together with Lifeact-mCherry under the *Neuralized* promoter. Arrowheads, cortical Actin. Note that for *dsh*^*RNAi*^ (**a**, **b**, **e**) only SOPs with polarity defects (reflected by the Pon signal) were selected (less than 10% penetrant phenotype) and for *Gβ13F*^*RNA*^ (**d**), only symmetrical cells were selected for the averaged linescans (n = 13). **e)** Projected area ratio of daughter-cells ($\frac{{{{{\mathrm{Area}}}}_{{{{\mathrm{Posterior}}}}\,{{{\mathrm{PIIA}}}}}}}{{{{{\mathrm{Area}}}}_{{{{\mathrm{Anterior}}}}\,{{{\mathrm{PIIB}}}}}}}$); Kruskal-Wallis non-parametric one-way ANOVA followed by Dunn’s post hoc test (P < 0.001). Note that the Control and the *naus*^*RNAi*^ data are shown again from Fig. 2b. **f)** Raw (unbinned) data, showed in Fig. 2g, of Posterior cortical Actin enrichment $\Delta {{{\mathrm{I}}}} = \frac{{(I_2 - I_1)}}{{(I_1 + I_2)}}$ *versus* Daughter-cell size ratio ($\frac{{{{{\mathrm{Area}}}}_{{{{\mathrm{Posterior}}}}}}}{{{{{\mathrm{Area}}}}_{{{{\mathrm{Anterior}}}}}}}$) in *mud*^*4*^ mutant. Dashed line, linear Fit. **g**, **h)** Time-lapse spinning disk confocal imaging of a SOP expressing WAVE-GFP (green) under the *Neuralized* promoter (**g**) and its associates linescan (**h**) across the dashed white arrows (20-pixel width linescan centered around the white dashed lines) shown in **g**. **i)** Phalloïdin staining (yellow; far-red probe) of SOPs expressing WAVE-GFP (green), Lifeact-mCherry (red) and the nanobody GBP-Pon under the *Neuralized* promoter. The anterior targeting of WAVE generates an anterior Lifeact crescent (both F-Actin and G-Actin specific) colocalizing with the Phalloïdin staining (only F-Actin). **j)** Signal intensity along linescans from SOPs expressing WAVE-GFP, Lifeact-mCherry (red) and the nanobody GBP-Pon under the *Neuralized* promoter to visualize Lifeact (red; left panel) and WAVE (green; right panel). **k)** Maximum projection of SOPs expressing Lifeact-CRY2^FL^-mCherry (red) and CIB^1^-GFP-WAVE and UAS-*lgl*^*3A*^ under the *Neuralized* promoter. In the control (upper panel), CRY2^FL^/CIB^1^ interaction has not been activated by blue light and therefore CIB^1^-GFP-WAVE is not targeted to one pole by optogenetics. Optogenetic targeting (lower panel) of CIB^1^-GFP-WAVE is achieved by polar illumination with blue light (dashed box) at anaphase onset. Timing of blue light illumination is indicated. Note that while in *lgl*^*3A*^ conditions, SOPs divide with symmetric size, targeting of WAVE to the one pole leads to asymmetric daughter size. **l)** Daughter-cell projected areas (posterior/anterior) in conditions shown in **k**. T-Test two-tailed (P < 0.001). **m)** Maximum projection of SOPs expressing WAVE-GFP, Lifeact-mCherry and the nanobody GBP-Baz under the *Neuralized* promoter. **n)** Signal intensities along linescans from SOPs expressing WAVE-GFP (green), Lifeact-mCherry (red) and GBP-Baz under the *Neuralized* promoter to visualize Lifeact (red; lower panel) and WAVE (green; upper panel). All data are presented as mean values +/− SEM unless stated otherwise. *n* = number of cells from 3, 4, 16, 7, 5, 2 pupae for **b**, **d**, **f**, **h**, **j**, **n** respectively, from 10, 3, 7, 8, 3 pupae for **e** and 11, 5 pupae for **l** from left to right respectively. Scale bars: 5 µm. Source numerical data are available in source data. Source data

While in control SOPs the polarity marker Pon is restricted to the anterior cortex by the PAR complex^39^ (Figs. 1a and 2a), in *lgl*^*3A*^ mutants, Pon is found at the cell cortex of both poles^38^ (Fig. 2a). Strikingly, in *lgl*^*3A*^, SOPs divide into two daughters of similar size (Fig. 2b) and exhibit symmetric cortical Actin (Figs. 1g and 2c,d; see also Extended Data Fig. 5a–e for *dsh* and *Gβ13F* phenotypes). This indicates that the mechanism underlying the posterior accumulation of Actin is under the control of the PAR complex. To further study the relationship between Actin and size asymmetry, we next uncoupled Actin polarity from the position of the mitotic plane.

### Polar Actin asymmetry levels correlate with daughter size

During SOP mitosis, three features align to the anterior–posterior axis: (1) the posterior cortical PAR domain (excluding Pon to the anterior), (2) the mitotic spindle orientation and (3) the posterior cortical Actin domain. A Mud–Dynein complex couples spindle orientation to the PAR domain^40^. Indeed, in *mud*^4^ mutants, the Pon domain is bisected in random locations by the cleavage plane^41–43^ (Fig. 2e). In this condition, daughter-cell sizes display a broad range from being asymmetric as in wild type to symmetric daughters (Fig. 2b).

Like in control SOPs, the Actin and Pon domains in *mud*^*4*^ still exclude each other, confirming that Actin is enriched within the PAR domain (Fig. 2e,f), consistent with Par6/Cdc42 interactions as previously reported^44–46^. Consequently, not only the Pon domain but also the Actin domain is randomly partitioned between the two poles in *mud*^*4*^ (Fig. 2e,f). This condition therefore generates a continuum of cortical Actin asymmetry levels that correlate with the degree of cell size asymmetries (Fig. 2g and Extended Data Fig. 5f): low levels of Actin asymmetry give rise to symmetric sizes, while posterior enrichment of Actin leads to wild-type*-*like asymmetric daughter-cell size. Therefore, the level of Actin asymmetry ultimately forecasts the relative sizes of the two daughters.

### Nanobody mistargeting of Actin can invert size asymmetry

**Fig. 3:**
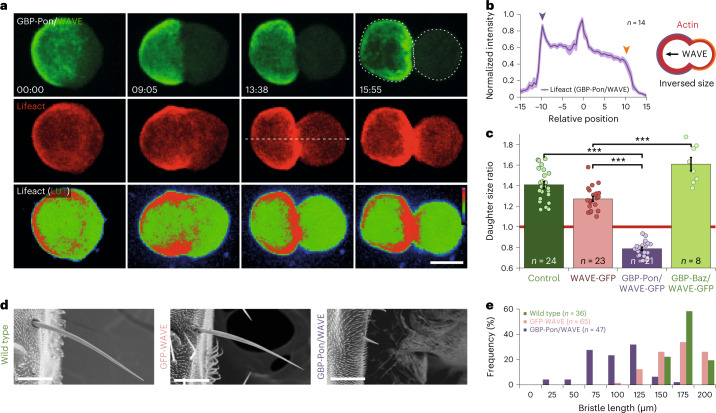
Cortical Actin manipulation through polar nanobodies. **a**, Maximum projection of SOPs expressing WAVE-GFP (green), Lifeact-mCherry (red) and GBP-Pon, an anti-GFP nanobody fused to the localization domain of Pon, under the *Neuralized* promoter. Note the anterior enrichment of WAVE-GFP (green) and Actin (red). At the end of division (last timepoint, 15:55), inversed asymmetric cell size. Note that, conversely, targeting Myosin with the same approach leads to a smaller rather than a larger daughter cell (Extended Data Fig. 4h–l). Scale bar, 5 µm. **b**, Signal intensity along linescan (20-pixel width linescan centred around the white dashed line) of Lifeact-mCherry; example of linescan shown in **a**. Mean ± s.e.m. **c**, Ratio of daughter-cell projected areas (posterior/anterior) in SOPs expressing Lifeact-mCherry (control), together with WAVE-GFP, and in conditions of WAVE-GFP mistargeting to the anterior pole by means of the GBP-Pon nanobody (GBP-Pon/WAVE-GFP) or to the posterior pole by means of the GBP-Bazooka nanobody (GBP-Baz/WAVE-GFP) (mean ± s.e.m.; Kruskal–Wallis one-way ANOVA on ranks, followed by a Dunn’s post hoc test, *P* < 0.001). **d**, Scanning electron microscopy images of post-vertical bristles of wild type (left), WAVE overexpression (GFP-WAVE, middle) and WAVE mistargeting to the anterior pole by means of the GBP-Pon nanobody (GBP-Pon/GFP-WAVE, right). Scale bars, 50 µm. **e**, Bristle lengths distribution in wild type (green), control (GFP-WAVE alone; pink) and GBP-Pon/GFP-WAVE (violet). *n* indicates number of cells from 9 pupae in **b**, from 6, 10, 9 and 3 pupae in **c** for control, WAVE-GFP, GBP-Pon/WAVE-GFP and GBP-Baz/WAVE-GFP measurements, respectively, or *n* indicates number of bristles in **e** from 18, 33 and 24 adults *Drosophila* for wild type, GFP-WAVE and GBP-Pon/GFP-WAVE measurements, respectively. Source numerical data are available in source data. Source data

In *mud*^*4*^ experiments, the position of the PAR domain and the Actin domain remain correlated (Fig. 2e,f): PAR might control in parallel Actin asymmetry and size asymmetry, where size would be Actin independent. To study whether polar Actin can directly control size, we inverted the Actin asymmetry with nanobodies^47,48^ while keeping the PAR polarity normal. We co-expressed in SOPs a GFP fusion of WAVE, the major cortical regulator of Arp2/3 (ref. ^49^) (Extended Data Fig. 5g,h), and GFP-binding protein (GBP)-Pon, an anti-GFP nanobody fused to the localization domain of Pon. This way, GFP-tagged WAVE is targeted to the anterior cortex, causing an anterior cortical accumulation of branched F-Actin (Fig. 3a,b and Extended Data Fig. 5i,j) and daughter-cell size inversion: the anterior daughter becomes larger than the posterior (Fig. 3a,c and Supplementary Video 2). Interestingly, while, in *lgl*^*3A*^ mutant, daughter-cell size is symmetrical, targeting WAVE to one pole by optogenetics (see below for details) in *lgl*^*3A*^ mutant is sufficient to yield this pole larger and can thereby generate asymmetric daughter-cell size (Extended Data Fig. 5k,l).

In contrast, targeting Myosin, α-Actinin or Fimbrin (two crosslinkers generating F-Actin bundles^34–36^) by nanobody targeting to the anterior pole leads to a smaller rather than a larger daughter cell (Extended Data Fig. 4f–l and Supplementary Information III.II.2.c). This indicates that the effect of branched Actin on the cell cortex mechanics is distinct from Myosin-mediated contractile tension (see below).

Conversely, we exacerbated Actin asymmetry beyond wild type by targeting WAVE-GFP to the posterior cortex through co-expression of GBP-Baz^48^, a GBP fusion to the *Drosophila* Par3 orthologue Bazooka. This leads to a more extreme size asymmetry compared with control SOPs (Fig. 3c and Extended Data Fig. 5m,n). These nanobody WAVE experiments suggest that the relative daughter-cell sizes can be directly determined by the cortical Actin asymmetry. In wild type, PAR controls Actin asymmetry and Actin, in turn, determines unequal cell size.

Interestingly, in the inversion experiment in which PIIA becomes smaller than PIIB (Fig. 3a,c), we noticed that bristle length in the adult fly was also reduced (Fig. 3d,e). This bristle cell derives from the lineage of the large PIIA cell, opening the possibility that PIIA/PIIB size asymmetry impacts the final cell sizes in the subsequent lineage and thereby the functions of these mechanoreceptors.

### Impairing cortical branched Actin at the cell poles

In the previous experiments, we enriched the cortical Actin in each pole. We next impaired the cortical Actin asymmetrically through targeting of Arpin, AIP1 or Cofilin. Arpin inhibits Arp2/3 (ref. ^50^), while AIP1 or Cofilin sever Actin filaments^51^. Posterior targeting of these factors using GBP-Baz caused embryonic lethality precluding their study at the subsequent pupal stage. To overcome this, we established optogenetics for temporal control based on CRY2/CIB^52^, whose binding is induced by 445/488 nm laser light.

**Fig. 4:**
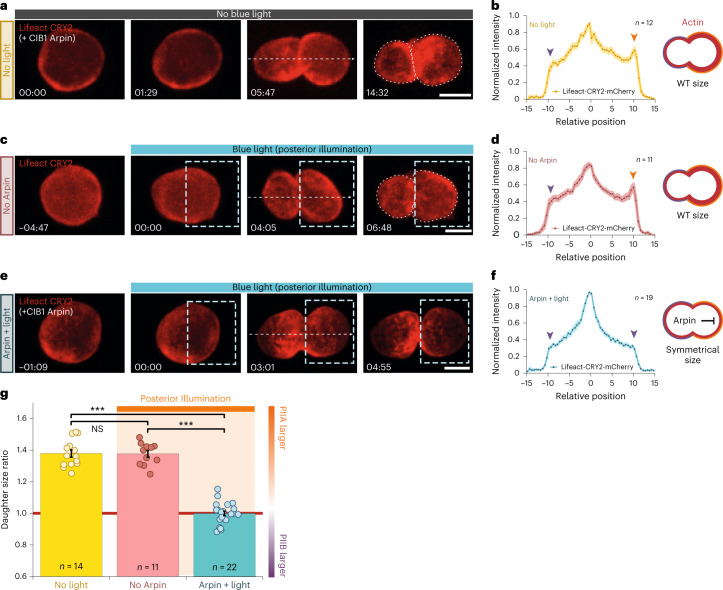
Impairing cortical branched Actin at the posterior pole through optogenetics leads to symmetric daughters. **a**–**f**, Maximum projection (**a**,**c**,**e**) and corresponding linescans of the mCherry signal (**b**,**d**,**f**) of SOPs expressing Lifeact-CRY2^FL^-mCherry (red) and CIB^1^-GFP-Arpin (**a**,**b**,**e**,**f**) or Lifeact-CRY2-mCherry only (**c**,**d**) under the *Neuralized* promoter. In the control (**a**,**b**), CRY2^FL^/CIB^1^ interaction has not been activated by blue light and therefore CIB^1^-GFP-Arpin is not targeted posteriorly by optogenetics. In a second control (**c**,**d**), the CIB^1^-GFP-Arpin transgene is not present but blue light is applied to the ROI (dashed box). Optogenetic targeting (**e**,**f**) of CIB^1^-GFP-Arpin to the posterior cortex is achieved by posterior illumination with blue light (dashed box) at anaphase onset. Timing of blue light illumination is indicated. Note that asymmetric localization of Pon in the anterior pole and the mitotic spindle positioning (Extended Data Fig. 6f–i) are not affected in these conditions. *n* indicates number of cells from 10, 6 and 10 pupae for **b**, **d** and **f**, respectively. **g**, Daughter-cell projected areas (posterior/anterior) in conditions shown in **a**–**f**. Kruskal–Wallis non-parametric one-way ANOVA, followed by a Holm–Sidak test (*P* < 0.001). Scale bars, 5 µm. All data are presented as mean ± s.e.m. *n* indicates number of cells from 10, 6 and 10 pupae for ‘no light’, ‘no Arpin’, and ‘Arpin + light’, respectively. Source numerical data are available in source data. Source data

**Extended Data Fig. 6:**
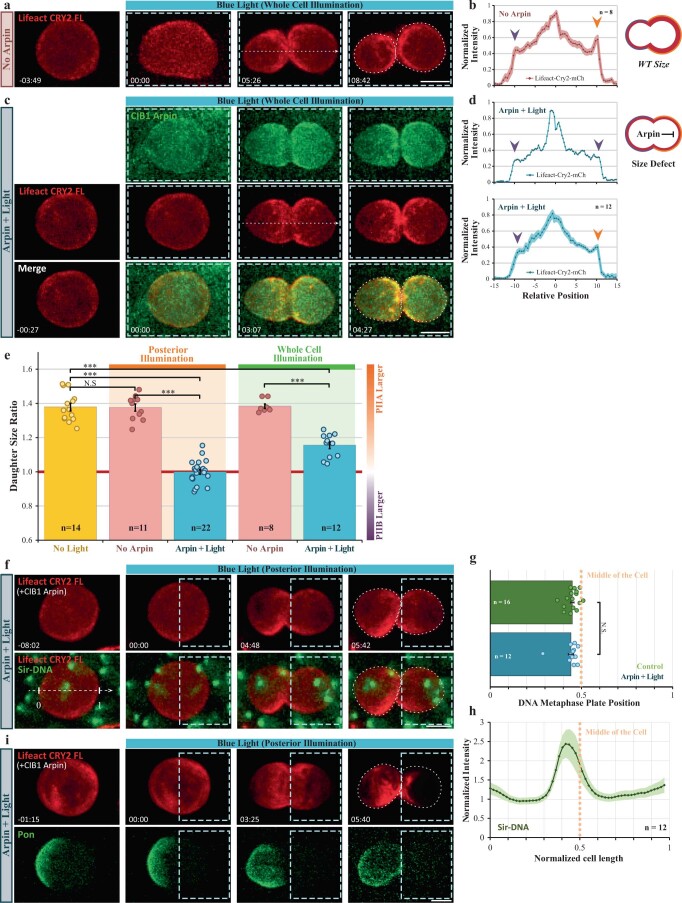
Impairing Actin asymmetry through optogenetics leads to symmetric daughters. **a)** Maximum projection of a SOP expressing Lifeact-CRY2^FL^-mCherry (Red) under the *Neuralized* promoter. **b)** Signal intensity of the Lifeact-mCherry channel from linescans along SOPs as in **a** (third timepoint). **c)** Maximum projection of a SOP expressing Lifeact-CRY2^FL^-mCherry (Red) and CIB^1^-GFP-Arpin under the *Neuralized* promoter. Upon blue light illumination of the whole cell in anaphase A (second timepoint), CRY2^FL^ and CIB^1^ interact leading to the recruitment of Arpin into Actin rich regions. **d)** Signal intensity of the Lifeact-mCherry channel from linescans along the SOP showed in **c** (upper panel) or averaged linescans (n=12; lower panel). Note the loss of the cortical Actin enrichment at the posterior pole (**d**), ultimately leading to symmetric daughter-cell size (**c**). **e)** Daughter-cell size ratio (projected areas ratio, Kruskal-Wallis non-parametric one-way ANOVA followed by Dunn’s post hoc test, P < 0.001). Note that ‘No Light’ and ‘Posterior Illumination’ data are shown again from Fig. 4g. **f)** Maximum projection of an SOP expressing Lifeact-CRY2^FL^-mCherry (Red) and CIB^1^-GFP-Arpin under the *Neuralized* promoter together with SiR-DNA probe (green). **g)** DNA metaphase plate (probed by SiR-DNA) positions as shown in **f**. The middle of the cell has been positioned to 0.5 highlighted by the dashed line. Mann-Whitney Rank Sum Test two-sided, N.S. **h)** Averaged signal intensities from linescans across SOPs in metaphase incubated with SiR-DNA, as shown in **f**. **i)** Maximum projection of a SOP expressing Lifeact-CRY2^FL^-mCherry (Red), CIB^1^-GFP-Arpin and FP670-Pon (green) under the *Neuralized* promoter. Note that the asymmetric localization of Pon (cell polarity marker) in the anterior pole is not affected upon optogenetic targeting of Arpin in the posterior pole. All data are presented as Mean ± SEM. *n* = number of cells from 3, 5, 4 pupae for **b**, **d**, **h** respectively and from 10, 6, 10, 3, 5 pupae for **e** from left to right and 4, 4 pupae for **g** from top to bottom respectively. Scale Bar 5 µm. Source numerical data are available in source data. Source data

By co-expression of Lifeact-CRY2^FL^-mCherry and CIB^1^-GFP-Arpin in SOPs, we recruited Arpin to Actin-rich regions with spatio-temporal control (posterior cortex at anaphase onset) using holographic patterned photo-stimulation microscopy^53^. Under these conditions, the relative Actin asymmetry between the two poles is lost (Fig. 4a–f and Supplementary Video 3) and daughter-cell sizes become symmetrical (Fig. 4g). As expected, when Arpin is optogenetically targeted to both poles, only the general levels of Actin density are affected and the effect on size asymmetry is observed to a lesser extent (Extended Data Fig. 6a–e). Furthermore, when size asymmetry is abolished by impairing branched F-Actin nucleation at the posterior cortex, cell polarity and, in particular, asymmetric spindle positioning are not affected (Extended Data Fig. 6f–h). This suggests that asymmetric spindle positioning by itself is not enough to drive size asymmetry (see below).

**Extended Data Fig. 7:**
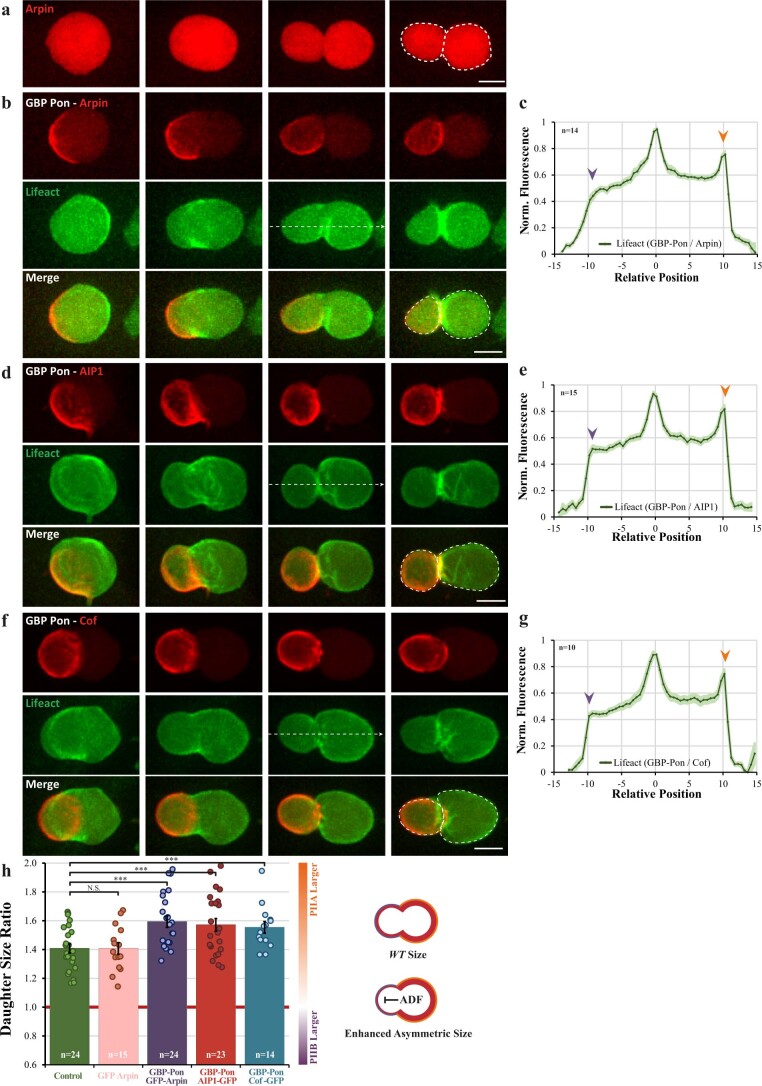
Nanobody mistargeting of Actin Depolymerizing Factors (ADF). **a)** Maximum projection of SOP expressing GFP-Arpin under the *Neuralized* promoter. Note the cytosolic localization of the Arpin signal. **b**-**g)** Maximum projection of SOP expressing Lifeact-mCherry (green), GBP-Pon and GFP-Arpin (red) (**b**), AIP1-GFP (Flare-GFP in *Drosophila*, an Actin depolymerizing factor) (**d**) or Cofilin-GFP (Drosophila Cofilin, an Actin filament severing factor) (**f**) under the *Neuralized* promoter. Corresponding signal intensities showed in **c**, **e**, **g** from linescans across SOPs in late anaphase to visualize the fluorescence signal observed in **b**, **d**, **f**. Scale bars 5μm. **h)** Daughter-cell size ratio (projected areas ratio). The co-expression of the nanobody GBP-Pon together with Actin Depolymerizing Factors leads to an enhancement of the daughter-cell size asymmetry (One-way ANOVA test followed by a Holm-Sidak post hoc test, P < 0.001). All data are presented as mean values +/− SEM. Note that ‘Control’ data are shown again from Fig. 3c. *n* = number of cells from 8, 7, 4 pupae for **c**, **e**, **g** respectively and 8, 5, 8, 7, 4 pupae for **h** from left to right respectively. Source numerical data are available in source data. Source data

Conversely, impairing the cortical Actin meshwork only in the anterior pole by using GBP-Pon and GFP fusions to Arpin, AIP1 or Cofilin led to a small but notable enhancement of daughter size asymmetry (Extended Data Fig. 7a–h). Furthermore, while inhibiting Formins does not affect size asymmetry, downregulating branched Actin nucleation leads to symmetrical daughters (Fig. 2b and Extended Data Figs. 2c–f and 5e), confirming that it is branched Actin that could modulate the daughter-cell sizes.

Taken together, our results confirm that the relative amount of branched Actin between the two cortices and, in particular, posterior branched Actin enrichment are key to producing size asymmetry.

### Polar Actin acts beyond spindle positioning

**Extended Data Fig. 8:**
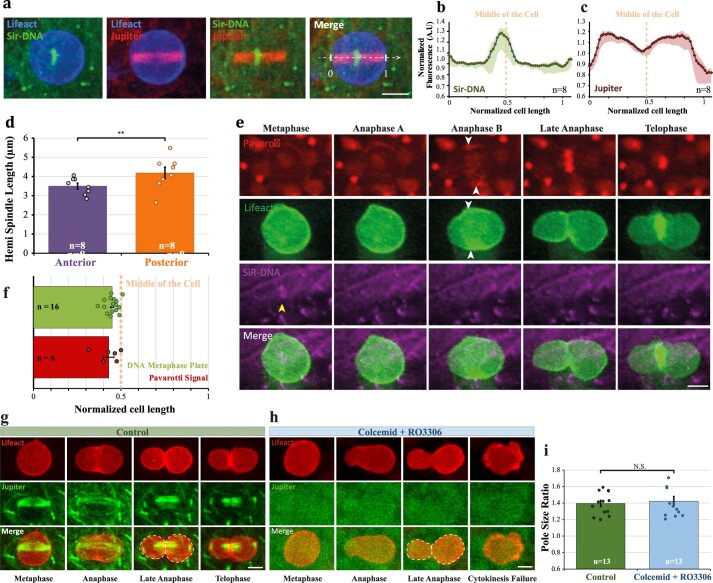
Asymmetric positioning of the mitotic spindle does not govern daughter-cell sizes. **a)** MAX-Projection of a metaphase SOPs expressing Jupiter-GFP (microtubules; red) and Lifeact-mCherry (blue) together with the far-red probe SiR-DNA (green). **b**, **c)** Averaged signal intensities from linescans across SOPs in metaphase tagged with SiR-DNA (**b**) or expressing Jupiter-GFP (**c**). The middle of the cell has been positioned to 0.5 highlighted by the dashed line. **d)** Hemi-spindle length measurements in metaphase SOPs (Mann-Whitney Rank Sum Test two-sided, P < 0.01). **e)** Maximum projections of time-lapse spinning disk confocal imaging of SOP expressing Lifeact-mCherry (green), Pavarotti-GFP (red) together with the far-red probe SiR-DNA (magenta). **f)** DNA metaphase plate and central spindle complex (probed by Pavarotti-GFP) positions. From geometry constrains only, this mild asymmetry of spindle positioning (**b-d**) leading to the mild asymmetry of the actomyosin ring positioning (**e-f**) is not sufficient to explain the final asymmetry of daughter sizes observed during SOP division (See Supplementary Information II.II). **g**, **h)** Maximum projection of SOP expressing Lifeact-mCherry (Red) under the *Neuralized* promoter together with endogenous Jupiter-GFP (microtubule; green) incubated without (**g**) or with (**h**) the microtubule depolymerizing drug Colcemid (+ Roscovitine to bypass the metaphase-arrest checkpoint). Note the disappearance of the microtubule signal in the Colcemid conditions. **i)** Pole size area ratios between control and cells treated with Colcemid + Roscovitine to depolymerize all microtubules (T-Test two-tailed, N.S.). All data are presented as mean values +/− SEM. *n* = number of cells from 3 pupae for **b**, **c**, **d** respectively, 4, 2 pupae for **f** from top to bottom and 4, 4 pupae for **i** from left to right respectively. Scale Bar 5μm. Source numerical data are available in source data. Source data

Cortical actomyosin is known to influence mitotic spindle positioning^54^. The antiparallel microtubule overlap established in metaphase positions the centralspindlin complex, which itself organizes the actomyosin ring in anaphase^55,56^ and, hence, the cytokinetic furrow^2,57^. This could, in turn, determine daughter-cell size. In SOPs, the metaphase plate is asymmetrically positioned towards the anterior pole (Extended Data Fig. 8a–f), yet this asymmetry is not enough to explain the asymmetry of size observed in wild type (Supplementary Information II.II). Furthermore, SOPs treated with Colcemid, to depolymerize all microtubules, still generate two poles with a size asymmetry similar to wild-type SOPs (Extended Data Fig. 8g–i), as previously reported in *Drosophila* neuroblasts^14,15^. This further supports the fact that asymmetric spindle positioning does not dominate unequal cell size in SOPs. Nonetheless, this prompted us to ask whether the inversion of daughter-cell size observed during the anterior WAVE targeting could be influenced by an Actin-mediated spindle positioning process during metaphase or whether it was only dependent on the polar Actin cortex having an effect on cortical mechanics.

**Fig. 5:**
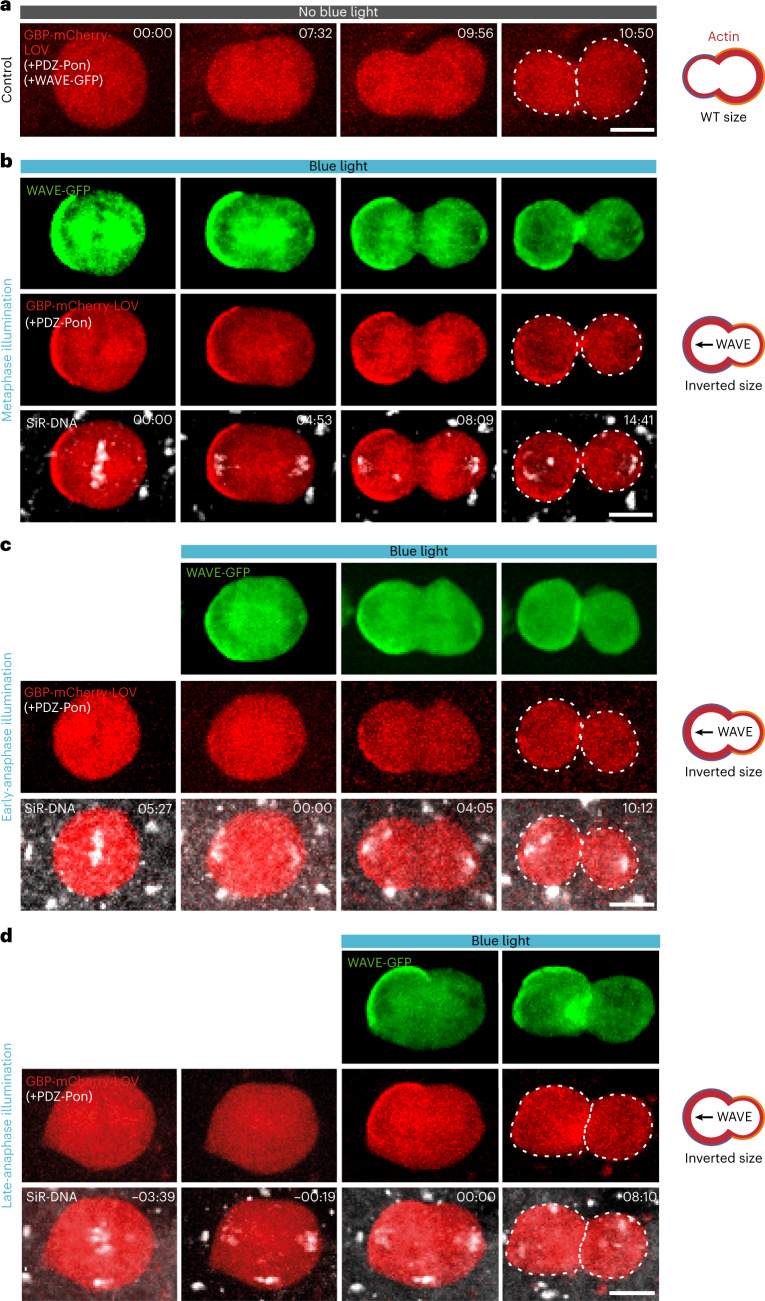
Late anaphase manipulation of cortical Actin leads to daughter-cell size inversion. **a**–**d**, Maximum projection of SOPs expressing the opto-nanobody GBP-mCherry-LOV (red), WAVE-GFP (green) and PDZ-Pon (unlabelled) under the *Neuralized* promoter without (**a**) or upon blue light illumination of the whole cell to target WAVE-GFP to the anterior cortex at different stage of cytokinesis (**b**–**d**). In **b**–**d**, SiR-DNA far-red probe (white) defines the mitotic phase. Illumination timing (timepoint 00:00) is indicated. Upon blue light illumination in metaphase (**b**), in early anaphase (**c**) or late anaphase (**d**), the complex LOV-mCherry-GBP/WAVE-GFP is targeted into the anterior cortex and SOPs thus divide with an inversed daughter-cell size asymmetry. Note that anterior enrichment of branched Actin, up to late anaphase, leads to daughter-cell size inversion. Scale bars, 5 µm.

To test this, we used LOV/PDZ optogenetics, where blue illumination opens the LOV domain to expose a PDZ binding domain^58^, at different stages of cytokinesis. We co-expressed three proteins: WAVE-GFP, GBP-LOV and PDZ-Pon (Fig. 5a–d). WAVE-GFP binds GBP-LOV and, upon blue illumination, is targeted to PDZ-Pon in the anterior cortex (Fig. 5b–d). Photoactivation at any timepoint from metaphase to late anaphase led to cell size inversion through Actin enrichment in the anterior cortex (Fig. 5b–d and Supplementary Video 4). Importantly, in late anaphase, the positions of the spindle midzone and the actomyosin ring are already committed (Extended Data Fig. 8e,f). Yet, photoactivation in late anaphase still affected daughter size asymmetry (Fig. 5b). Therefore, polar cortical Actin itself can control daughter size asymmetry independently of spindle positioning and relative daughter-cell sizes can still be modified until late stages of cytokinesis. This is consistent with the possibility that cortical Actin directly controls cell size by regulating the material properties and mechanics of the polar cortex itself.

**Extended Data Fig. 9:**
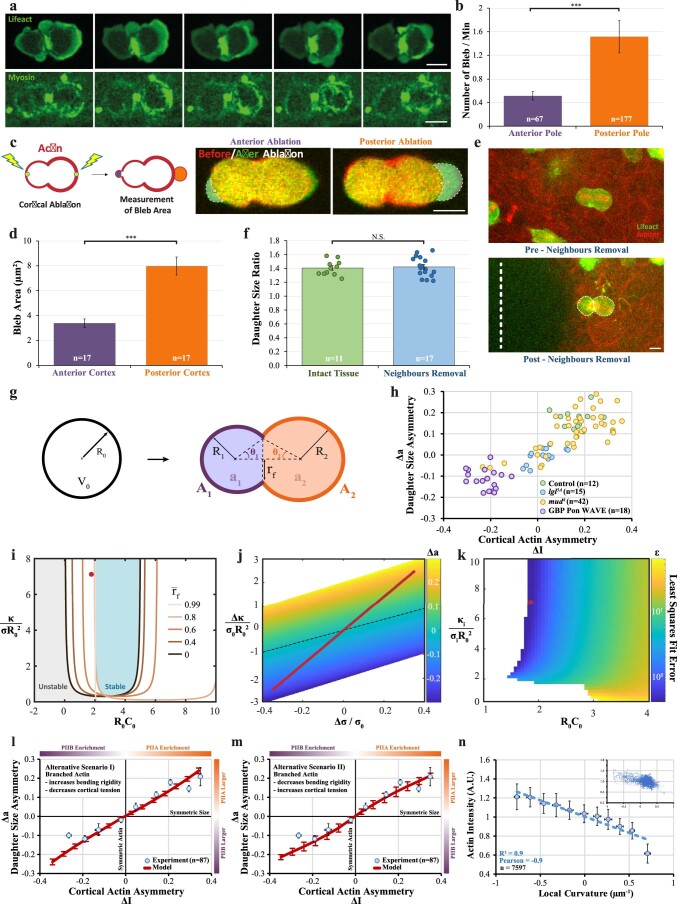
Asymmetric cortical mechanics, parameterization of surface shapes through spherical caps and mechanical shape stability. **a)** Time-lapse spinning disk confocal imaging of SOPs at late stages of cytokinesis expressing Lifeact mCherry (upper panel) or endogenous Spaghetti Squash (lower panel). We could observe accumulation and bursts of Myosin at the end of the cytokinesis (mainly in the posterior pole) due to blebbing: Myosin has been shown to be required in the bleb retraction process^98–100^. **b)** Number of blebs per minute observed at the end of the division in the anterior and in the posterior poles of the dividing cells (Mean +/− SEM, Mann-Whitney Rank Sum Test two-sided, P < 0.001). **c)** Laser-induced bleb from laser ablation of the cell cortex. Snapshots of timepoints before (red) and after laser ablation (green). **d)** Laser-induced bleb area measurements from anterior cortex ablation and posterior cortex ablation (Mean +/− SEM, Mann-Whitney Rank Sum Test two-sided, P < 0.001). Blebs have been shown to be reporters of local cortical tension and their size to be proportionally correlated with cortical tension^59^. Therefore, this asymmetric blebbing (innate and induced) uncovers a higher effective tension in the posterior cortex. **e)** Asymmetric removal of the neighbour cells does not impact daughter-cell size during SOP division. Scale bar 5 µm. **f)** Daughter-cell size ratio between control (Intact Tissue) and tissue where all the neighbour cells anterior to the SOP have been removed by laser ablation (Neighbours Removal). This corroborates the idea that daughter size is controlled only by an intrinsic mechanism relying on the cell cortex. Mean +/− SEM, T-Test two-tailed, N.S. **g)** We consider surface shapes with fixed volume *V*_0_ and corresponding reference radius *R*_0_. Cell divisions are represented by two connected spherical caps that are characterized by corresponding radii *R*_*i*_, surface areas *A*_*i*_, opening angles *θ*_*i*_ and the furrow radius *r*_*f*_. The projected areas *a*_1_ and *a*_2_ (light shaded orange and violet regions) are used to compare model geometries to experimentally measured projected area asymmetries $\Delta a = \frac{{(a_2 - a_1)}}{{(a_1 + a_2)}}$. **h)** Daughter-cell size asymmetry Δa versus cortical Actin asymmetry $\Delta I = \frac{{(I_2 - I_1)}}{{(I_1 + I_2)}}$ (binned shown in Fig. 6b, c). **i)** Linear stability diagram of symmetric surfaces (*A*_1_=*A*_2_) with homogeneous material properties *σ*_1_=*σ*_2=_*σ*, *κ*_1_=*κ*_2_=*κ* and at different relative furrow radii $\overline {r_f} = r_f/R_0$. A symmetric position of the neck is stable for all furrow radii in the blue-shaded region. Red dot indicates best fit of the minimal model (see **i**, **k**). **j)** Shape asymmetries $\Delta a = \frac{{(a_2 - a_1)}}{{(a_1 + a_2)}}$ resulting from bending rigidity asymmetries $\Delta \kappa = \frac{{(\kappa _2 - \kappa _1)}}{2}$ and surface tension asymmetries $\Delta \sigma = \frac{{(\sigma _2 - \sigma _1)}}{2}$. Black dashed line depicts symmetric surface geometries ($\Delta a = 0$). Considering a fixed mean tension *σ*_*m*_, the mean bending rigidity was set to $\frac{{\kappa _m}}{{\sigma _mR_0^2}} = 7.1$. **k)** Least squares fit error of shape trajectories for different fit parameters $\frac{{\kappa _i}}{{\sigma _iR_0^2}} = \frac{{\alpha _\kappa }}{{\alpha _\sigma R_0^2}}$ (see Eqs. (12) and (13) from Supplementary Info. Theory) and *R*_0_*C*_0_ with the best fit shown in Fig. 6b, c. Intensity asymmetries $\Delta I = \frac{{(I_2 - I_1)}}{{(I_1 + I_2)}}$ translate to bending rigidities and surface tensions as described by Eqs. (12) and (13) (See Supplementary Info. Theory Section). The shape trajectory depicted in Fig. 6b, c (red line of the model) can therefore be represented in **j** as well (red solid line). In the white regions of **j** and **k** no (stable) equilibrium shapes exist or they represent improper constricted surfaces ($\theta _i > \pi /2$, see **g**). **l**, **m)** Theoretical shape curves for alternative scenarios that provide mechanical shape stability at early stages. I) Scenario in which increasing branched Actin density increases bending rigidity but decreases tension (**l**) (See Supplementary Info. Theory Section III.II.4). II) Scenario in which increasing Actin density increases tension but decreases local bending rigidity (**m**) (See Supplementary Info. Theory Section III.II.4). In both cases **l** and **m**, different pairs of Actin density (*I*_1_,*I*_2_), that yield the same values of normalized density difference $\Delta I = \frac{{(I_2 - I_1)}}{{(I_1 + I_2)}}$, can give rise to different projected area asymmetries $\Delta a = \frac{{(a_2 - a_1)}}{{(a_1 + a_2)}}$. Solid line and error bars depict the corresponding mean and standard deviation, respectively. **n)** Cortical Actin density versus local curvature along the cell contour. At the furrow region (curvature < 0), cortex highly enriched with Actin and Myosin at the actomyosin ring corresponds to negative curvature. At the cell poles (curvature ≥ 0), cortex with low cortical Actin show highly curved contours whereas cortex highly enriched with Actin corresponds to flatter contours (curvature = 0). Mean +/− StDev. Insert: Raw data. Source numerical data are available in source data. *n* = number of cells from 6, 6 pupae for **b**, **d** respectively, 6, 9 pupae for **f** from left to right respectively, 38 pupae for **h**, **l** and **m**, and 8 pupae for **n**. Source data

Indeed, mechanical properties between the two poles are different. Blebs are local reporters of cortical tension: their size correlates indeed with cortical tension^59^. At the end of SOP mitosis, blebs are more frequent and larger in the posterior pole (Extended Data Figs. 1e and 9a,b). We also confirmed this cortical tension asymmetry by generating laser-induced blebs by ablation of the cell cortex (Extended Data Fig. 9c,d).

### Physics of daughter-cell size asymmetry

We then established a minimal physical model of cortical mechanics to study (1) which mechanical factors, modulated by Actin, are required for asymmetric size, (2) whether differences in cortical Actin asymmetry levels can explain quantitatively the experimental size asymmetries observed and (3) under which conditions are daughter-cell shape geometries mechanically stable. Following experimental observations (Extended Data Fig. 9e,f), our model neglects effects from neighbouring cells.

**Fig. 6:**
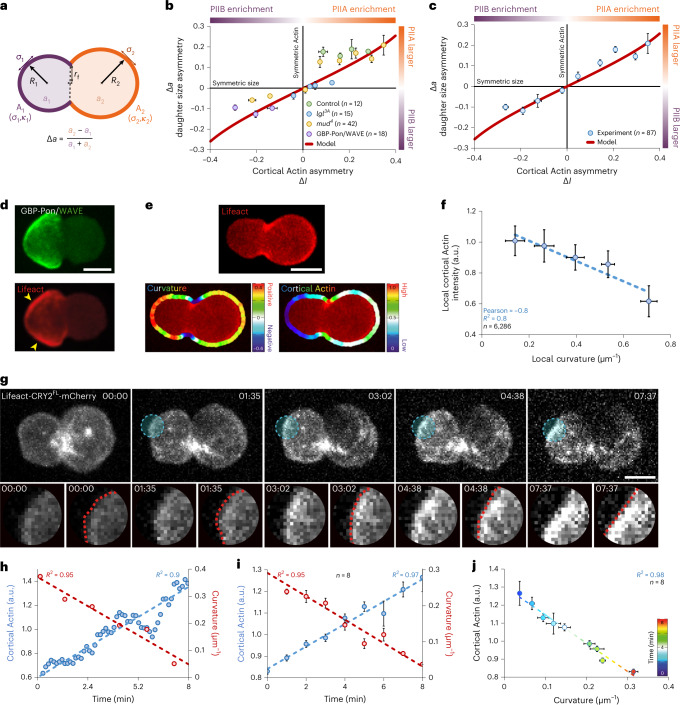
Physics of cell size asymmetry: branched Actin asymmetry controls daughter-cell size through curvature. **a**, Cell surface geometries during divisions are represented by two connected spherical caps characterized by their surface areas *A*_1_, *A*_2_ and their specific mechanical properties *σ*_1_, *σ*_2_ and *κ*_1_, *κ*_2_ corresponding to the cortical tensions and bending rigidities, respectively. *R*_1_, *R*_2_ denotes the radii and *r*_f_ the cleavage furrow radius. Our minimal model focuses on Actin-dependent material properties of the cortex to explain cell size asymmetries (Supplementary Information III). **b**,**c**, Daughter-cell size asymmetry Δ*a* ($\Delta {{a}} = \frac{{{{{\mathrm{Area}}}}_{{{{\mathrm{PIIA}}}}}-{{{\mathrm{Area}}}}_{{{{\mathrm{PIIB}}}}}}}{{{{{\mathrm{Area}}}}_{{{{\mathrm{PIIB}}}}} + {{{\mathrm{Area}}}}_{{{{\mathrm{PIIA}}}}}}} = \frac{{({{a}}_2 - {{a}}_1)}}{{({{a}}_1 + {{a}}_2)}}$) versus cortical Actin asymmetry Δ*I* ($\Delta {{I}} = \frac{{{{{I}}}_{{{{\mathrm{Posterior}}}}\,{{{\mathrm{cortical}}}}\,{{{\mathrm{Actin}}}}}-{{I}}_{{{{\mathrm{Anterior}}}}\,{{{\mathrm{cortical}}}}\,{{{\mathrm{Actin}}}}}}}{{{{I}}_{{{{\mathrm{Anterior}}}}\,{{{\mathrm{cortical}}}}\,{{{\mathrm{Actin}}}}} + {{I}}_{{{{\mathrm{Posterior}}}}\,{{{\mathrm{cortical}}}}\,{{{\mathrm{Actin}}}}}}} = \frac{{({{I}}_2 - {{I}}_1)}}{{({{I}}_1 + {{I}}_2)}}$) in experiments and theory. Data are presented as mean ± s.e.m. **d**, Maximum projection of SOP expressing WAVE-GFP, Lifeact-mCherry and the nanobody GBP-Pon under the *Neuralized* promoter. Yellow arrows, low curvatures of the cell contour where accumulation of cortical branched Actin is observed. **e**, Maximum projection of SOP expressing Lifeact-mCherry (top) and corresponding curvature along the cell contour and cortical Actin signal density, colour coded according to the LUT to the right (bottom). **f**, Cortical Actin density versus local curvature along the cell contour. At the cell poles (curvature >0), cortex with low cortical Actin show highly curved contours whereas cortex highly enriched with Actin corresponds to flatter contours. Mean ± standard deviation. **g**–**j**, Local cortical Actin optogenetics to manipulate curvature: maximum projection of a SOP expressing Lifeact-CRY2^FL^-mCherry (white) and CIB^1^-GFP-WAVE under the *Neuralized* promoter, where, upon blue light illumination in the ROI (blue dashed line), CRY2^FL^ and CIB^1^ interact leading to WAVE targeting into the illuminated cortical region (**g**); cortical Actin signal (blue) in the illuminated ROI and curvature (red) of the cell contour after blue light illumination within the circle ROI for the experiment shown in **g** (**h**) and an average of eight experiments (**i**); cortical Actin versus curvature within the illuminated ROI, with dashed lines indicating linear fits (**j**). Scale bars, 5 µm. Data are presented as mean ± s.e.m. unless stated otherwise. *n* indicates number of cells from 8, 5, 16 and 9 pupae in **b** for control, *lgl*^*3A*^, *mud*^*4*^ and GBP-Pon/WAVE, respectively, from 38 pupae in **c** and 8, 3 and 3 pupae for **f**, **i** and **j**, respectively. Source numerical data are available in source data. Source data

We considered a minimal model where a spherical cell of radius *R* divides into two daughter cells, represented by two connected spherical caps^60^ (Fig. 6a and Extended Data Fig. 9g) that can have different mechanical surface properties (Supplementary Information III): a contractile cortical tension *σ*, a bending rigidity *κ* (cortical stiffness in response to bending deformations) and a cortical spontaneous curvature *C*_0_ (intrinsic tendency to bend). The cortical actomyosin cytoskeleton could modulate these material properties^61,62^, thereby contributing to cortical mechanical responses, cortical curvature and, ultimately, relative daughter sizes.

We explored a mechanism where *κ* and *σ* are determined by the cortical Actin density *I*. For simplicity, both *κ* and *σ* increase linearly with *I*, according to *κ* *=* *α*_*κ*_*I* and *σ* *=* *α*_*σ*_*I* (Supplementary Information III.II.3). In this model, different Actin asymmetry levels between the two caps, for given *α*_*κ*_, *α*_*σ*_ and *C*_0_ values, give rise to different force-balanced surface geometries characterized by a size asymmetry ∆*a* (Fig. 6a). Consequently, we fit our model to the experimentally measured, Actin-dependent PIIA/PIIB cell size asymmetries (*R*^2^ = 0.96; Fig. 6b,c and Extended Data Fig. 9h).

For the fit, only two free parameters need to be determined: (1) the ratio *α*_*κ*_/*α*_*σ*_ and (2) *C*_0_ (relative to cell size; Supplementary Information III.II.3). With these two parameters alone, we can quantitatively explain the cell size ratios observed in our experiments, from the asymmetric wild-type scenario and the symmetric mutant conditions to those in which the size ratios are inverted (Fig. 6b,c). Importantly, the fairly large value of $\frac{{\alpha _\kappa }}{{\alpha _\sigma R^2}} = 7.1 \pm 0.8$ obtained from the fit suggests that Actin-mediated modulations of cortical bending rigidity, as characterized by *α*_*κ*_, play a crucial role in ACD and that changes in cortical Actin density have a much stronger effect on bending rigidity than on cortical tension (Extended Data Fig. 9i–k). This reveals a critical role of cortical Actin in determining daughter-cell sizes during ACD by providing substantial cortical bending elasticity.

Formin-mediated Actin is considered as a key determinant of cell mechanics^63^ through its impact on cortical contractility^64^. We show, however, that branched Actin modulates cortical mechanics to control size asymmetry in SOPs. This uncovers two fundamental aspects in cell mechanics: (1) branched cortical Actin can also play a key role for cell mechanics, and (2) not only contractility but also bending rigidity is important for cell morphogenesis.

Finally, we studied the mechanical stability of the two emerging spherical caps during furrow constriction. If the system is unstable, the furrow slips to one side: the smaller cap collapses into the bigger one. For the fit parameters obtained above, the system is least stable at the beginning of cytokinesis but stabilizes after 25% constriction (Extended Data Fig. 9i and Supplementary Information III.II.2). A reliable constriction during early cytokinesis can be achieved, for example, if constriction is faster than the collapse of the smaller cap or if branched Actin decreases cortical contractility^65^ (Extended Data Fig. 9l,m and Supplementary Information III.II.4).

### Cortical Actin determines size by modulating local curvature

Our theoretical model suggests that local changes in Actin density modulate the mechanical properties of the cell cortex locally to induce local shape changes of the cell contour. Indeed, when WAVE is mistargeted to the anterior cortex with nanobodies, local enrichment of branched Actin in patches correlates with a local cell contour flattening (Fig. 6d). To investigate this further, we measured the local enrichment of cortical Actin and the local cell curvature in normal conditions (Fig. 6e,f). At the cell poles, Actin density in the cortex correlates with curvature (Fig. 6f and Extended Data Fig. 9n). This suggests that Actin density itself could tune the cell contour curvature by modulating local cortical mechanics: flattening the cell surface contributes to the emergence of a larger daughter cell.

We then looked at the curvature dynamics while locally targeting WAVE in real time to the cortex though optogenetics (Fig. 6g–j and Supplementary Video 5). Figure 6i shows that cortical flattening upon local branched Actin enrichment happens in only a few minutes. These fast dynamics are compatible with a role of cortical Actin to shape cells during cytokinesis, which occurs at a timescale of about 15 min.

## Discussion

**Extended Data Fig. 10:**
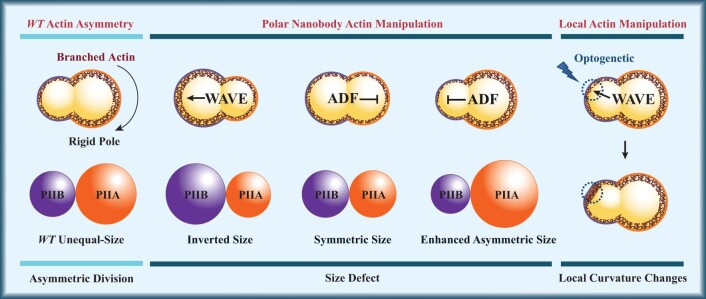
Summary of observations to manipulate daughter-cell sizes during asymmetric cell division. Summary of main observations. An asymmetric cortical Actin is observed in *wild-type* with more branched Actin in the posterior pole. When Actin asymmetry is inversed (that is, through WAVE nanobody targeting to promote branched Actin polymerization in the anterior cortex), cell size inversion is observed. Conversely, when Actin asymmetry is impaired in the posterior pole (that is, through *lgl*^*3A*^ overexpression or Actin-Depolymerization Factors (ADF) nanobody targeting to inhibit Actin polymerization), symmetric daughter-cells is observed. When Actin asymmetry is enhanced (that is, through ADF nanobody targeting to inhibit Actin polymerization in the anterior pole), an enhancement of the asymmetric size between the two daughter-cells is observed. Local branched Actin enrichment at the cell cortex, through optogenetic WAVE recruitment, was leading to lower curvatures of the cell contour.

Our work shows that polarized cortical Actin produces unequal-size daughters during ACD. In particular, branched Actin modulates the cytokinetic cell shape by locally setting the bending rigidity, and thereby determines the daughter-cell size asymmetry (for summary, see Extended Data Fig. 10a). This is based on the following key observations: (1) In wild type, cortical branched Actin accumulation is observed in the larger posterior pole (Fig. 1f,g) and local Actin density correlates with local curvature (Fig. 6d–f and Extended Data Fig. 9n). (2) When cortical Actin is symmetrical, the SOP divides with symmetric daughter sizes (Figs. 1g, 2b–d and 4e–g and Extended Data Figs. 2c,d, 5a–e and 6c–e). (3) When cortical Actin asymmetry is inverted, cell size is inverted (Figs. 3a–c and 5b–d). (4) When cortical branched Actin polymerization is induced locally by optogenetics, the cortex flattens locally (Fig. 6g–j).

During SOP division, an asymmetry in bending rigidity is sufficient to achieve asymmetric sizes (Extended Data Fig. 9i–k). Our theoretical approach shows that an Actin dependence of both bending rigidity and tension accounts quantitatively for the observed correlation between cortical Actin density and daughter-cell size in all experimental conditions (Fig. 6b,c). However, our data show that Actin impacts bending rigidity much more strongly than tension: cortical branched Actin hence sets the bending rigidity of the cell cortex. This uncovers a distinctive, fundamental role of bending rigidity, instead of cortical tension, for asymmetric daughter-cell size determination.

In systems such as the *Drosophila* and *Caenorhabditis* neuroblasts, cortical tension due to Myosin asymmetries has been proposed as a major player for generating asymmetric daughter-cell sizes^5,14,15^. Here we propose that the modulation of daughter-cell sizes can be mediated by two mechanisms, each related to a specific population of Actin: linear Actin together with Myosin leading to tension asymmetries and branched and crosslinked Actin leading to bending rigidity asymmetries. However, the control of bending rigidity by branched Actin, which we discovered here, adds to the toolkit to generate stable ACDs with unequal daughter-cell sizes. Finally, our findings may have implications for the understanding of cortical mechanics during stem cell divisions, which are not only important for cell shape and tissue morphogenesis, but also for cell fate assignation and tumour growth.

## Methods

### Key resources table

Materials, reagents and resources used in this study can be found in Supplementary Table 1.

### Experimental model and subject details

#### Fly handling, fly lines and maintenance

All fly stocks were maintained at 18 °C. Fly crosses were raised at 25 °C, then kept at 16 °C until pupariation, and shifted to different temperature depending on the experiments (25 °C or 29 °C as indicated in Supplementary Table 1). To express genes in the SOPs, the *Neuralized* promoter together with the UAS-Gal4 system was used. We also used the Gal80^ts^ protein to reduce the levels of expression. For example, we used Gal80^ts^ to achieve low levels of Zipper (Myosin Regulatory Heavy Chain) expression to prevent motor aggregates (Extended Data Fig. 4e) or to prevent embryonic lethality in *Gβ13F*^*RNAi*^.

##### Transgenes used in this study

UAS-Cofilin::GFP (this study), UAS-GBP::mCherry::LOV (this study), UAS-GFP::Arpin (this study), UAS-CIB^1^::Arpin (this study), UAS-PDZ::Pon (this study), UAS-CIB^1^::WAVE (this study), UAS-GFP::Cdc42 (this study), UAS-GFP::WAVE (this study), UAS-Lifeact::CRY2^FL^::mCherry (this study), UAS-Lifeact::mCherry (this study), UAS-FP670::Pon (this study), Ubi-mCherry::Pavarotti^48^ (Gonzalez Lab), UAS-GBP::Pon^48^ (Gonzalez Lab), UAS-GBP::mCherry::Pon^48^ (Gonzalez Lab), UAS-GBP::Bazooka^48^ (Gonzalez Lab), UAS-Sqh^E20E21^::GFP^69^ (gift from Thomas Lecuit), UAS-GFP::Utrophin^ABD^ (ref. ^70^) (gift from Thomas Lecuit), UAS-WAVE::GFP^71^ (gift from Sven Bogdan), UAS-GFP::Fascin^72^ (gift from Francois Payre), UAS-Dia::GFP^73^ (gift from Eduardo Moreno), UAS-Flare::GFP^74^ (gift from Paul N. Adler), UAS-DsRed (gift from François Karch), UAS-Zipper::GFP^75^ (gift from Andrea Brand), Zipper::GFP^76^ (Flytrap CC01626), Cheerio::GFP^77^ (Bloomington 60261), GFP::Rac1 (Bloomington 52285), Jupiter::GFP (Bloomington 6836), Tub-Gal80ts (Bloomington 7017), mud^4^ (ref. ^41^) (Bloomington 9563), Sqh::GFP (Bloomington 57144), Fimbrin::GFP (Bloomington 51562), α-Actinin::GFP (Bloomington 51573), UAS-*nausicaa*^*RNAi*^ (ref. ^24^) (VDRC 31375), UAS-*dsh*^*RNAi*^ (ref. ^78^) (VDRC 101525), UAS-Gβ13F^RNAi^ (ref. ^79^) (VDRC 31257), UAS-mRFP::Pon^80^, Neur-Gal4 (ref. ^81^), Ubi-GFP::Pavarotti^82^, UAS-GFP::Pon^83^, UAS-*lgl*^*3A*^ (ref. ^84^).

More details on the transgenes used can be found in Supplementary Table 1.

#### Detailed genotypes and temperatures

Detailed genotypes and temperatures can be found in Supplementary Table 1.

#### Neuroblast culture

Live imaging was performed as described previously^85^. Briefly, brains were dissected in collagenase buffer and incubated in collagenase for 15 min (0.2 mg ml^−1^ collagenase, Sigma C0130). Brains were manually dissociated into a culture medium (Schneider’s medium supplemented with glucose, fetal calf serum, fly extract and insulin) as in ref. ^85^.

Fluorodish was first activated using a plasma cleaner (Harrick Plasma, PDC-32G) for 3 min and then coated with poly-l-lysine. Dissociated brains were transferred onto the activated and coated Fluorodish. Cells were allowed to settle and adhere to the coverslip 30 min before imaging.

#### Collagenase buffer composition

The composition of 10× collagenase buffer were as follows: 8 g NaCl (Panreac 131659.1211), 0.2 g KCl (Merck TA 52 0336), 0.05 g NaH_2_PO_4_ (Merck 13799), 1 g NaHCO_3_, 1 g d(+)-glucose (Merck 20174 312) and *quantum satis* 100 ml H_2_O and add 2 mg ml^−1^ collagenase.

#### Schneider glucose supplemented composition

Schneider’s medium (Gibco 21720-024) supplemented with 1 mg ml^−1^ glucose (Merck 20174 312).

#### Neuroblast culture medium composition

The composition of 2.5 ml of Schneider + glucose medium were as follows: 300 µl FBS (10%, Thermo Fisher #10270106), 75 µl fly extract (inactivated and filtered sterilized medium: 100 g of smashed Oregon flies into 680 ml of cold M3 insect medium, Millipore Sigma, #S3652), 3 µl insulin (Sigma 19278) and 30 µl P/S (Gibco 15140).

#### Plasmids

Most of the open reading frames cloned by PCR for this study were flanked by FseI and AscI (FA) sites for convenient shuttling between compatible plasmids.

- UAS-GFP::WAVE: For GFP N-ter WAVE cloning, the *Drosophila* WAVE coding region was digested by FA from the plasmid: pCS2 GFP dWAVE, gift from Alexis Gautreau, and inserted into a *Drosophila* UAS expression plasmid pUAST4 PC GFP FA blue.
- UAS-GFP::Arpin: Similarly, for GFP N-ter Arpin cloning, Arpin coding region was digested by FA from the plasmid pcDNA5 FRT His PC TEV Arpin, gift from Alexis Gautreau^50^, and inserted into a *Drosophila* UAS expression plasmid pUAST4 PC GFP FA blue. N‐terminal GFP tagging of Arpin has been previously shown to be functional.
- UAS-GBP::mCherry::LOV: We also cloned the GBP, or so-called GFP nanobody, a lama VHH single-chain antibody against GFP for expression of fusion proteins in the fly with Light Oxygen Voltage Sensing Domain (LOV), a photo-interacting protein able to interact with a PDZ domain from *Avena sativa*^58^. The GBP nanobody has been previously cloned into a pUAST4 GBP FA blue plasmid as described in ref. ^48^. mCherry-LOV, flanked with FA restriction sites, is a synthetic gene to remove unwanted AscI sites inside the sequence (IDT). We also added a flexible linker (glycine- and serine-rich linker) between mCherry and the LOV peptide. It was then inserted in C-ter into pUAST4 GBP FA blue plasmid.
  For the synthetic mCherry::LOV gene and oligonucleotide sequences used, see Supplementary Table 1.
- UAS-PDZ::Pon: ePDZb1, flanked with EcoR1 and FseI (EF) restriction sites, is a synthetic gene to remove unwanted EcoR1 sites inside the sequence (IDT). It was then inserted in N-ter into pUAST4 EF Pon^LD^ plasmid, where Pon^LD^ is the Pon Localization Domain (corresponding to amino acids 474–670 of the Pon protein^83^) cloned from w^1118^ flies cDNA into a pUAST4 *Drosophila* plasmid as described in ref. ^48^.
  For the synthetic PDZ gene and oligonucleotide sequences used, see Supplementary Table 1.
- UAS-Lifeact::mCherry: Lifeact was amplified by PCR, flanked with FA, from pIRES puro Lifeact mCherry plasmid, gift from Guillaume Montagnac, and inserted into a pUAST4 FA mCherry plasmid.
  For oligonucleotide sequences used, see Supplementary Table 1.
- UAS-Lifeact::CRY2^FL^::mCherry: For the full-length CRY2 protein cloning, mCherry-CRY2^FL^ was amplified by PCR, flanked with AscI and NotI (AN) digestion sites from the plasmid available from Addgene collection ID 26871 (pCRY2^FL^deltaNLS-mCherryN1) and was inserted in C-ter into pUAST4 FA Lifeact AN mCherry plasmid pre-digested by AN for replacement of mCherry by CRY2^FL^-mCherry.
  For oligonucleotide sequences used, see Supplementary Table 1.
- UAS-CIB^1^::GFP::Arpin: CIB^1^-GFP was amplified by PCR, flanked with EF, from plasmids available from Addgene collection ID 28240 (pCIB^1^deltaNLS-pmGFP) and was inserted in N-ter into pUAST4 EF GFP FA Arpin plasmid pre-digested by EF for replacement of GFP by CIB^1^-GFP.
  For oligonucleotide sequences used, see Supplementary Table 1.
- UAS-CIB^1^::GFP::WAVE: WAVE FA insert was isolated and purified from pUAST4 PC GFP FA WAVE and inserted into a pUAST4 CIB^1^ GFP FA Arpin plasmid pre-digested by FA for replacement of Arpin by WAVE.
- UAS-GFP::Cdc42: For the Cdc42 protein cloning, Cdc42 was amplified by PCR, flanked with FA digestion sites from the plasmid available from Addgene collection ID 52248 (pUASp YFP Cdc42 WT) and was inserted in C-ter into pUAST4 PC GFP FA plasmid pre-digested by FA for insertion of the Cdc42 FA fragment. The plasmid was then was sent to BestGene to be injected into *w*^*1118*^ background *Drosophila* embryo to generate transgenic*s*.
  For oligonucleotide sequences used, see Supplementary Table 1.
- UAS-FP670::Pon: Similarly to the UAS-PDZ::Pon cloning above, FP670 synthetic gene (IDT), flanked with EF restriction sites, was then inserted in phase and in N-ter into pUAST4 EF Pon^LD^ plasmid. The plasmid was then was sent to BestGene to be injected into *w*^*1118*^ background *Drosophila* embryo to generate transgenic*s*.
  For the synthetic FP670 gene sequence, see Supplementary Table 1.
- UAS-dCofilin::GFP: dCofilin corresponds to the *Drosophila* Twinstar protein. For dCofilin::GFP, a synthetic gene has been created with a GFP inserted in the middle, between N74 and G75, surrounding by 12 AA flexible linkers (GSA) on each side of GFP and with a STOP codon at the end. The synthetic gene has been flanked by FA sites. Post FA digestion, it was then inserted in N-ter into pUAST4 FA mCherry plasmid. For the synthetic Cofilin::GFP gene sequence, see Supplementary Table 1.

All the cloning has been confirmed by sequencing (Fasteris). All synthetic genes have been generated by IDT (G-blocks gene fragment). Injection of plasmids into *Drosophila* embryos to generate transgenics was performed by BestGene in *w*^*1118*^ background.

### Imaging method details

#### Fly notum imaging

Fly notum dissection and SOP imaging was performed in clone 8 medium after embedding into a fibrinogen clot in order to diminish tissue movements during fast 3D image acquisition as described^86^. Imaging was performed using a 3i Marianas spinning disk confocal setup based on a Zeiss Z1 stand, a 63× PLAN APO NA 1.4 objective and a Yokogawa X1 spinning disk head followed by a 1.2× magnification lens and an Evolve EMCCD camera (Photometrics). Fast *z*-stack acquisition of entire SOP cells (0.4 µm steps) was obtained using a piezo stage (Mad City Labs). Single-emitter emission filters were always used to avoid bleed-through, and each channel was acquired sequentially. To increase acquisition speed, we acquired 3D stacks spanning only 16–18 µm along the *z* axis, which is usually sufficient to contain the entire dividing SOP. To assure linearity and avoid saturation, fluorescent signal was systematically checked to be within the camera dynamic range. Unless stated otherwise, data presented in figure panels correspond to maximum-intensity projections.

#### Clone 8 medium composition

The composition for 250 ml were as follows: 6.5 ml fly extract (inactivated and filtered sterilized medium: 100 g of smashed Oregon flies into 680 ml of cold M3 insect medium, Millipore Sigma, #S3652), 5 ml of foetal bovine serum (Thermo Fisher Scientific, #10270106), 125 µl insulin from bovine pancreas (Millipore Sigma, #I1882) and 238.62 ml Shields and Sang M3 Insect Medium (Millipore Sigma, #S3652).

#### SiR-DNA probe

To follow the different mitosis stages, after fibrinogen clot embedding described as above, clone 8 imaging media were supplemented with 1 μM SiR‐DNA^87^ (Spirochrome) and incubated at least 10 min at room temperature before imaging and left into the imaging media until the end of the experiment.

#### FRAP

FRAP of Actin-GFP (Extended Data Fig. 1k,l) was performed on the fly genotype *w*^*1118*^*; UAS-GFP-Actin 5C/+; Neur-Gal4/+* on the 3i Marianas spinning disk confocal setup based on a Zeiss Z1 stand, a 63× PLAN APO NA 1.4 objective and a Yokogawa W1 spinning disk head followed by a 1.2× magnification lens and an iXon EMCCD Andor camera equipped with Vector Photomanipulation hardware, a high-speed galvanometer-based point scanner, driven by Slidebook 6.0.

Two circular bleaching regions (2 µm diameter) were drawn onto the anterior and the posterior cortex and bleached simultaneously on both poles in late anaphase. Owing to the fast recovery of Actin-GFP (timescale of few seconds), recovery was monitored in 2D (one *z* plane) to maximize frame rate and ensure acquisition is as fast as possible. The recovery was then monitored by spinning disk confocal imaging at an average frame rate of 4.3 Hz.

For each FRAP experiment, we followed four different regions:

1. Anterior FRAP region of interest (ROI);
2. Posterior FRAP ROI;
3. Photobleaching ROI: cytoplasmic ROI within the same cell to measure the decay in fluorescence due to the acquisition bleaching;
4. Background ROI: ROI outside the cell to measure the offset intensity.

The photobleaching and background curves have been fitted with single exponential functions of the form:

$$I\left( t \right) = A + Be^{ - \frac{{(t - t_0)}}{\tau }}$$

Anterior and posterior FRAP curves were first processed for signal background subtraction (with the ROI outside the cell) and for photobleaching correction (with the cytoplasmic ROI within the cell) as follows:

$$I_{{\mathrm{Anterior}}\,{\mathrm{FRAP}}\,{\mathrm{corrected}}}\left( t \right) = I_{{\mathrm{Anterior}}\,{\mathrm{FRAP}}}\left( t \right) - I_{{\mathrm{Background}}}(t)$$

$$I_{{\mathrm{Posterior}}\,{\mathrm{FRAP}}\,{\mathrm{corrected}}}\left( t \right) = I_{{\mathrm{Posterior}}\,{\mathrm{FRAP}}}\left( t \right) - I_{{\mathrm{Background}}}\left( t \right)$$

$$I_{{\mathrm{Photobleaching}}\,{\mathrm{corrected}}}\left( t \right) = I_{{\mathrm{Photobleaching}}}\left( t \right) - I_{{\mathrm{Background}}}(t)$$

FRAP curves were then processed for photobleaching correction (with the cytoplasmic ROI within the cell) through double normalization^88^ as follows:

$$I_{{\mathrm{FRAP}}\,{\mathrm{double}}\,{\mathrm{norm}}}\left( t \right) = \frac{{I_{{\mathrm{Pre}} - {\mathrm{photobleaching}}\,{\mathrm{corrected}}}}}{{I_{{\mathrm{Pre}} - {\mathrm{FRAP}}\,{\mathrm{corrected}}}}}\frac{{I_{{\mathrm{FRAP}}\,{\mathrm{corrected}}}\left( t \right)}}{{I_{{\mathrm{Photobleaching}}\,{\mathrm{corrected}}}\left( t \right)}}$$

where *I*_Pre-FRAP norm_ and *I*_Pre-photobleaching norm_ correspond to the average pre-bleaching intensities in the FRAP ROIs and in the photobleaching ROI, respectively.

Additionally, we performed full-scale normalization according to the following formula:

$$I_{{\mathrm{FRAP}}\,{\mathrm{double}}\,{\mathrm{norm}}\,{\mathrm{full}}\,{\mathrm{scale}}}\left( t \right) = \frac{{I_{{\mathrm{FRAP}}\,{\mathrm{double}}\,{\mathrm{norm}}}\left( t \right) - I_{{\mathrm{FRAP}}\,{\mathrm{double}}\,{\mathrm{norm}}}\left( {{\mathrm{bleach}}} \right)}}{{I_{{\mathrm{Pre}} - {\mathrm{FRAP}}\,{\mathrm{double}}\,{\mathrm{norm}}} - I_{{\mathrm{FRAP}}\,{\mathrm{double}}\,{\mathrm{norm}}}\left( {{\mathrm{bleach}}} \right)}}$$

where *I*_FRAP double norm_ (bleach) is the intensity value just after bleaching and *I*_Pre-FRAP double norm_ the average intensity before bleaching of the double normalized data.

Both anterior and posterior normalized FRAP curves were then fitted to a double exponential equation (as previously established for Actin dynamics^89,90^):

$$I_{{\mathrm{FRAP}}\,{\mathrm{double}}\,{\mathrm{norm}}\,{\mathrm{full}}\,{\mathrm{scale}}}\left( t \right) = A_1\left( {1 - e^{ - \frac{{(t - t_0)}}{{\tau _1}}}} \right) + A_2\left( {1 - e^{ - \frac{{(t - t_0)}}{{\tau _2}}}} \right)$$

In Extended Data Fig. 1l, the represented turnover is the average of *τ*_1_ and *τ*_2_. For the anterior pole *τ*_1_ = 0.3 ± 0.08; *τ*_2_ = 9.01 ± 0.9 and for the posterior pole *τ*_1_ = 0.8 ± 0.32; *τ*_2_ = 28.31 ± 2.2 and in Extended Data Fig. 1k, immobile fractions are: anterior IF =0.212 ± 0.06; posterior IF = −0.005 ± 0.05.

To fully describe the Actin turnovers at the cell cortex, the curves should additionally be corrected for a cytoplasmic diffusive recovery process as described in ref. ^91^. Yet, our FRAP experiment aims to compare the anterior and the posterior cortex dynamics, and we assume that this process is similar in both the anterior and the posterior cytoplasm.

#### Fly notum immunofluorescence

Fly notum staining was performed as previously described^86^: In brief, fly nota were dissected in PEM (80 mM PIPES, 5 mM EGTA and 1 mM MgSO_4_), then fixed during 20 min in PEM with 4% PFA (Electron Microscopy Science) followed by a 20 min incubation in PEM with 4% PFA and 0.2% Triton X-100. Nota were then processed for immunofluorescence using standard techniques.

For Actin filament visualization, we used Phalloidin 425 (from Sigma) at a 1/40 dilution. Coverslips were mounted in Prolong Gold anti-fade reagent (Molecular Probes). Image acquisition setting was then performed on the 3i Spinning disk confocal microscope W1 setup described above.

#### Drug treatments

To inhibit Formins, we incubated our tissue with 40 µM SMIFH2 drug.

To dissasemble spindles during division, we used the microtubule depolymerizing drug Colcemid at 10 µg ml^−1^ together with Roscovitine (CDK inhibitor) at 25 µM to bypass the metaphase-arrest checkpoint.

#### Nanobody targeting

In this study, we used the ‘nanobody assay’ established in ref. ^48^, consisting of a GBP (or so-called GFP nanobody), a lama VHH single chain antibody against GFP^47^ fused with polarity proteins in the fly (GBP::Pon and GBP::Bazooka). GBP::Pon will segregate in the anterior cortex as the wild-type Pon protein, whereas GBP::Baz will segregate in the posterior cortex.

Flies co-expressing GBP::Pon and WAVE::GFP (Fig. 3a–c) displayed occasional spindle orientation defects reflected by a division plane orthogonal to the polarity. Cells showing such spindle orientation defects were excluded from subsequent analysis.

We also used Gal80^ts^ to achieve lower levels of GBP-Baz to prevent ectopic expression of Bazooka (Extended Data Fig. 5m,n).

#### Photo-interacting protein optogenetic experiments

##### Whole-cell illumination

Whole-cell photomanipulation in Fig. 5a,b and Extended Data Figs. 6a–d and 8j,k was performed on the 3i Marianas spinning disk X1 setup described above as a stack acquisition with 488 laser at 250–500 ms and 405 laser at 10–20 ms exposure time to promote the interaction between the LOV peptide and the PDZ sequence protein or the CRY2^FL^ and CIB^1^ protein couple.

To target WAVE into the anterior pole upon light illumination, we used the *w; UAS-Pon::PDZ, UAS-GBP::mCherry::LOV/+; Neur-Gal4/UAS-WAVE::GFP* fly genotype (Fig. 5a,b and Extended Data Fig. 8j,k). We also performed the same experiment with C-ter GFP-tagged WAVE instead of N-ter and obtain similar results of Actin recruitment into the anterior cortex in *w; UAS-Pon::PDZ, UAS-GBP::mCherry::LOV/+; Neur-Gal4/UAS-GFP::WAVE* fly (data not shown).

To target Arpin into cortical Actin-rich regions for branched Actin inhibition upon light illumination, we used *w; UAS-CIB*^*1*^*::GFP::Arpin/+; UAS-Lifeact::mCherry::CRY2*^*FL*^*, Neur-Gal4/+* fly genotype (Extended Data Fig. 6c,d).

##### Posterior illumination

Posterior illumination in Fig. 4c–g and Extended Data Fig. 6f–i was performed on a 3i Marianas spinning disk confocal W1 setup described above with 3i Phasor hardware, a spatial light modulator-based computer-generated holography, driven by Slidebook 6.0. We generated a squared ROI centred in the middle of the cell in *z* englobing the posterior compartment to promote the interaction between the CRY2^FL^ and CIB^1^ protein couple within the posterior pole only. We used the Phasor 445 laser at 15–20% laser power for 500 ms exposure starting in anaphase onset and illuminated the region after each 3D stack acquisition. To target Arpin into the posterior cortex for branched Actin inhibition upon light illumination, we used the *w; UAS-CIB*^*1*^*::GFP::Arpin/+; UAS-Lifeact::mCherry::CRY2*^*FL*^*, Neur-Gal4/+* fly genotype (Fig. 4e–g and Extended Data Fig. 6f–h) or *w;* *UAS-CIB*^*1*^*::GFP::Arpin/UAS-FP670::Pon; UAS-Lifeact::mCherry::CRY2*^*FL*^*, Neur-Gal4/+* fly genotype (Extended Data Fig. 6i).

##### Local illumination

Local illumination in Fig. 6g–j was performed on a 3i Marianas spinning disk confocal W1 setup described above with the 3i Vector hardware. We used the vector 488 laser to illuminate a 3 µm circle ROI located in the anterior pole, the illumination ROI was centred in the middle of the cell in *z* for 50 ms to 100 ms exposure starting at the second timepoint and then performed after each 3D stack acquisition. We always located our ROI regions in the anterior pole for two reasons: (1) to avoid the influence of the posterior Actin enrichment observed in wild type and (2) because, in the smaller anterior pole, the curvature is higher and thus any contribution of branched Actin on the curvature of the cell cortex would have a more dramatic/visible effect in the anterior pole.

To target WAVE and promoting branched Actin polymerization into the small, illuminated ROI region at the cell cortex, we use the *w; UAS-CIB*^*1*^*::GFP::WAVE /+; UAS-Lifeact::mCherry::CRY2*^*FL*^*, Neur-Gal4/+* fly genotype (Fig. 6g–j).

#### Laser ablation

##### Cortex ablation

Cortical laser ablation was performed on the 3i Marianas spinning disk setup described above (63× NA 1.4 oil objective) equipped with Vector coupled with Ablate! pulsed laser ablation hardware driven by Slidebook 6.0 (Extended Data Fig. 9c,d). To cut the cortex, laser ablation was performed on SOPs expressing *w;; UAS-Lifeact::mCherry, Neur-Gal 4/+*. We performed sequential cuts: first in the anterior cortex and then in the posterior cortex. As we observed bigger blebs (innate and laser-induced) in the posterior pole, we always performed ablation of the cortex at the anterior pole first in order to discard the effect of an intracellular pressure release at the first ablation, which could induce bigger blebs. In the quantification, we selected only cells that were not damaged by the ablation (where laser-induced blebs could retract after ablation and SOPs were able to finish cytokinesis). The bleb areas were measured when the bleb sizes reached their maximum size before retraction.

##### Neighbour cell ablation

Laser ablation was performed on the 3i Marianas spinning disk setup as described above in nota expressing *w;; UAS-Lifeact::mCherry, Jupiter::GFP, Neur-Gal 4/+* (Extended Data Fig. 9e,f). We performed tissue ablation on entire pupae as described in ref. ^92^. To remove the impact of neighbour cells on the daughter-cell size asymmetry, we performed large cuts in the anterior side of dividing SOPs (in anaphase) to remove neighbour cells. We then measured daughter-cell size once cytokinesis was completed.

#### Scanning electron microscopy

Flies were killed by exposure to diethyl ether for 20 min, then mounted on scanning electron microscopy holders using double-sided carbon tape (Electron Microscopy Sciences) and subsequently treated with a gold sputter coater (JFC-1200, JEOL). Imaging was performed using a JEOL JSM-6510LV scanning electron microscope operating in high-vacuum mode using a working distance of 1 mm and an acceleration of 10–15 kV.

### Statistics and reproducibility

Unless stated otherwise, measurements are given as mean ± standard error of the mean (s.e.m.). All statistical analyses were performed using SigmaStat 3.5 software (Systat). Normality of variables was verified with Shapiro–Wilk tests. Homoscedasticity of variables was always verified when conducting parametric tests. In cases where variables failed normality and/or homoscedasticity tests, non‐parametric tests were applied.

Micrographs are representative of a set of at least two independent experimental rounds performed on different days and were in all cases reproducible. No statistical method was used to pre-determine sample size. The experiments were not randomized. The Investigators were not blinded to allocation during experiments and outcome assessment. As disclosed above in Methods, SOP cells showing spindle orientation defects were excluded from the WAVE nanobody experiment and, for the dshRNAi experiment, only cells with polarity defects were selected (low phenotype penetrance). For more detail, see Reporting Summary.

### Supplementary Note 1

For additional methods, in particular, on quantification and image analysis, please refer to section Supplementary Methods.

For additional information, please refer to section Supplementary Information.

For the theory, please refer to section Supplementary Theory.

### Reporting summary

Further information on research design is available in the Nature Portfolio Reporting Summary linked to this article.

## Online content

Any methods, additional references, Nature Portfolio reporting summaries, source data, extended data, supplementary information, acknowledgements, peer review information; details of author contributions and competing interests; and statements of data and code availability are available at 10.1038/s41556-022-01058-9.

## Supplementary information

Supplementary Information Supplementary methods, information, theory and references.

Reporting Summary

Supplementary Table 1 Key resources table. Detailed genotypes and temperatures. Oligonucleotides. Synthetic gene constructs.

Supplementary Video 1 Actin asymmetry during SOP division. SOP expressing Lifeact-mCherry (red) under the *Neuralized* promoter. Posterior enrichment of cortical Actin can be observed in late anaphase. Right: look-up table (LUT); redder pixels correspond to high fluorescence signals. Scale bar, 5 µm.

Supplementary Video 2 Inversion of Actin asymmetry leads to inversion of daughter-cell size. SOP expressing WAVE-GFP (green), Lifeact-mCherry (red) and GBP-Pon, an anti-GFP nanobody fused to the localization domain of Pon under the *Neuralized* promoter. Upon nanobody targeting of WAVE-GFP to the anterior pole, an enrichment of Actin (red) is visible, leading at the end of the division to an inversed asymmetric cell size. Scale bar, 5 µm.

Supplementary Video 3 Impairing cortical branched Actin at the posterior pole through optogenetics leads to symmetric daughters. SOP expressing Lifeact-CRY2^FL^-mCherry (red) and CIB^1^-GFP-Arpin under the *Neuralized* promoter. Optogenetic targeting of CIB^1^-GFP-Arpin to the posterior cortex by posterior illumination with blue light (blue box) at anaphase onset leads to the loss of the posterior Actin enrichment and ultimately to symmetrical daughter cell. Scale bars, 5 µm.

Supplementary Video 4 Late-anaphase manipulation of cortical Actin leads to inversion of daughter-cell size. SOP expressing the opto-nanobody GBP-mCherry-LOV (red), WAVE-GFP (green) and PDZ-Pon (unlabelled) under the *Neuralized* promoter together with the SiR-DNA far-red probe (white) to define mitotic phases. Upon blue light illumination, in late anaphase, LOV and PDZ interact leading to WAVE targeting into the anterior pole. Anterior enrichment of branched Actin through optogenetics, up to late anaphase, leads to daughter-cell size inversion. Scale bars, 5 µm.

Supplementary Video 5 Local branched Actin optogenetics to manipulate cell curvature. SOP expressing Lifeact-CRY2^FL^-mCherry (white) and CIB^1^-GFP-WAVE under the *Neuralized* promoter. Upon blue light illumination in the small circle ROI (blue circle), CRY2^FL^ and CIB^1^ interact leading to WAVE targeting in the illuminated cortical region. Branched Actin accumulates at the cortex and the cell contour becomes flatter in the ROI region. Scale bars, 5 µm.

## Data Availability

Source data are provided with this paper. All other data supporting the findings of this study are available from the corresponding author on reasonable request.

## Source data

Source Data Fig. 1 Statistical source data.
Source Data Fig. 2 Statistical source data.
Source Data Fig. 3 Statistical source data.
Source Data Fig. 4 Statistical source data.
Source Data Fig. 6 Statistical source data.
Source Data Extended Data Fig. 1 Statistical source data.
Source Data Extended Data Fig. 2 Statistical source data.
Source Data Extended Data Fig. 3 Statistical source data.
Source Data Extended Data Fig. 4 Statistical source data.
Source Data Extended Data Fig. 5 Statistical source data.
Source Data Extended Data Fig. 6 Statistical source data.
Source Data Extended Data Fig. 7 Statistical source data.
Source Data Extended Data Fig. 8 Statistical source data.
Source Data Extended Data Fig. 9 Statistical source data.

## Code availability

Custom codes written in ImageJ and MATLAB are available from the corresponding author upon reasonable request. Local curvature has been measured by an adapted MATLAB script from ref. ^93^.
