# Supplementary material for: Polarized branched Actin modulates cortical mechanics to produce unequal-size daughters during asymmetric division
Source: Nat Cell Biol. 2023 Feb 6;25(2):235–45. doi: 10.1038/s41556-022-01058-9 (PMC9928585; doi:10.1038/s41556-022-01058-9)
Supplement: Supplementary file 1 — Supplementary methods, information, theory and references. [file 41556_2022_1058_MOESM1_ESM.pdf]

nature cell biology

Article

https://doi.org/10.1038/s41556-022-01058-9

Polarized branched Actin modulates cortical mechanics to produce unequal-size daughters during asymmetric division

In the format provided by the authors and unedited

Supplementary Information

Polarized branched Actin modulates cortical mechanics to produce Unequal-Size Daughters during Asymmetric Division

Alicia Daeden[1], Alexander Mietke[2–5], Emmanuel Derivery[1,6], Carole Seum[1], Frank Jülicher[3], Marcos Gonzalez-Gaitan[1]

[1] Department of Biochemistry, Faculty of Sciences, University of Geneva, Geneva, Switzerland

[2] Department of Mathematics, Massachusetts Institute of Technology, Cambridge, USA

[3] Max Planck Institute for the Physics of Complex Systems, Dresden, Germany

[4] Max Planck Institute of Molecular Cell Biology and Genetics, Dresden, Germany

[5] Center for Systems Biology Dresden, Dresden, Germany

[6] MRC Laboratory of Molecular Biology, Cambridge, UK

I. Supplementary Methods

Quantification and Image Analysis

Unless otherwise specified, image analysis was performed using custom codes written in ImageJ and Matlab (Matlab 2019b," The MathWorks, Natick, MA, USA). For representation purposes, intensity was sometimes color-coded using lookup tables based on the Rainbow lookup tables in ImageJ (with red saturated pixels instead on grey).

Daughter Cell Geometry (Projected Area, Surface Area and Volume)

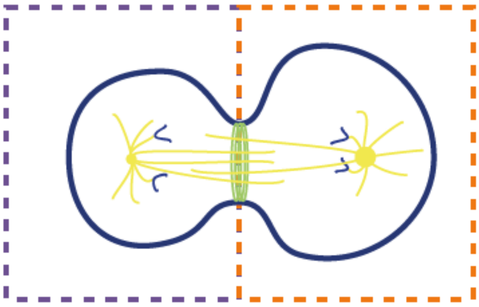

Volume measurement:

To observe and compare the daughter cell volumes, we used the 3D Counter Fiji Plugin on 3D stack acquisition of entire SOP cells expressing cytosolic GFP and RFP::Pon (to label the anterior pole). We measured the daughter cell volumes at the end of the cytokinesis, once the two-daughter cell are easily distinguishable and before the poles start to bleb (we describe the blebbing phenomena during SOP division in the main text). By focusing on this phase, we avoid the contribution of the bleb volumes in the measured daughter cell volume.

Projected Area measurement:

After a Maximum projection of the 3D stack, we measured the projected areas of the two daughter cells on the flies expressing cytosolic GFP. The anterior and the posterior poles have been delimited by a segmented line in ImageJ/Fiji. We observed that the volume of the two daughter cells correlates with their projected area (Extended Data Fig. 1c). Therefore, in the main text, because the measurement of volumes requires a cytosolic fluorophore (such as GFP) removing possibly an important channel, projected areas measurements are used as a substitute for the volume measurements and finally as a proxy for the daughter cell sizes.

Cell Surface Segmentation and 3D Rendering

To determine the dynamics of volume and surface areas in wild-type SOPs (**Fig. 1c, e**), cell segmentation on 3D stacks was performed over the course of the cytokinesis. To this end, we imaged SOPs labelled with cytosolic GFP to delineate cell volume and RFP::Pon to identify the

anterior pole. We then used the ImageJ/Fiji plugin 'Squassh'[1] (using the built-in background removal, automatic intensity estimation, Poisson noise model, Gaussian PSF model with σ_{xy} = 0.39 and σ_z = 1.40 and region filter size 30px) to segment dividing SOP cells based on their cytosolic GFP signal.

The following image analysis steps were performed using a tailored pipeline developed in Matlab. First, the axis of the binary mask was rotated in three dimensions such that long axis of the dividing cell was parallel to the stack planes (referred to as xy-plane in the following). The resulting data was then further processed going at each time point through all stack planes along the z-axis. In particular, in each plane, we removed objects smaller than 10px (Matlab function `bwareaopen`), filled holes of remaining regions (Matlab function `imfill`), determine the number of connected regions present (Matlab function `bwconncomp`) and represented each region as Level-Set (Matlab function `bwdist`). Using a level set representation of regions in each plane, a surface triangulation was determined (Matlab function `isosurface`, see **Fig. 1b**) from which the total volume and surface area at each time point were computed for each cell.

Before division, only a single connected region is typically found in each xy-plane that contains the cell. At every time point, the level-set representations of the regions in xy-plane were tested for the number of maxima present. The occurrence of two distinct maxima in any of the planes corresponds to an ingression of the cell outline and indicates the onset of cytokinesis. For the following time points, positions of the two-level set maxima were used to determine in each xy-plane a set of points that approximates the cross-section of the division plane with this particular xy-plane. This results at each time point in a set of coordinates distributed in 3 dimensions, to which a plane is fitted (see as an example the green plane in **Fig. 1b**). This plane is considered the division plane that splits the total surface area and cell volume determined previously into partial volumes and partial surface areas (purple and orange surfaces in **Fig. 1b**) associated with the anterior and the posterior pole. The first assignment of the pole identity during division was based on final cell sizes and cross-checked by the RFP::Pon signal co-localized with the segmented anterior surface.

To average the resulting temporal information of volumes and surface areas of each cell, the different time series were registered with respect to the first time point at which chromosomes could be seen split in the cytosolic GFP signal. Finally, measured volume and surface area data was for each cell normalized by the time-averaged volume $< V_0 >$ and by the reference area $A_0 = (4\pi)^{1/3}(3 < V_0 >)^{2/3}$, respectively, and the data of all cells (N=19) was binned in 24s intervals. The mean and standard deviation of the data in each bin is depicted in **Fig. 1c, e** of the main text by the solid lines and error bars, respectively.

Cortical Actin Asymmetry Quantification ("Averaged Linescans" Method)

Scheme of a SOP in anaphase illustrating the anterior (violet) and posterior cortical Actin (orange) as cited in the main text:

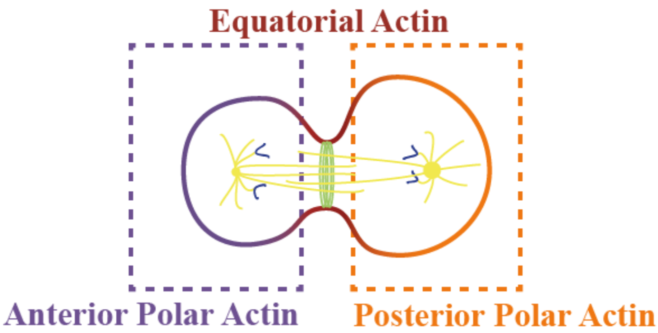

Maximum projection of SOP cells expressing Lifeact::mCherry with the Neuralized promoter have been selected to measure the cortical Actin intensities. During anaphase, for each pole (**Anterior** and **Posterior**), ten linescans have been acquired using a custom-designed ImageJ/FiJi macro.

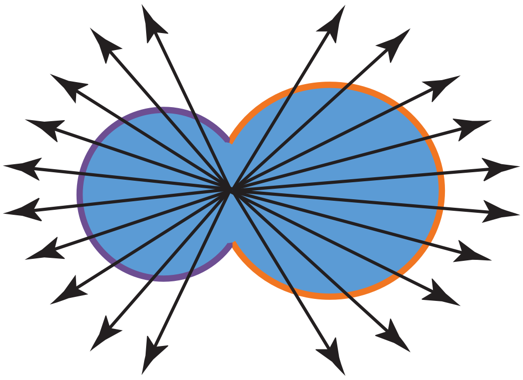

The cortical peak positions have been determined, usually with highest intensity, for each linescan. The extracellular background intensities have been determined by taking an average of fluorescence signals outside the cell (points to the right of the anterior and posterior cortical peak positions) and have been subtracted to obtain a corrected fluorescence signal. The intracellular background intensities have been determined by taking small circles (≈1.5µm of radius) inside each cell poles. We then averaged peak intensities for the anterior cortical Actin and the posterior cortical Actin, respectively:

$$\text{Anterior Cortical Actin} = \frac{1}{10}\sum_{i=1}^{10} [\text{Anterior Cortical Actin Peak}_{\ i}] - \text{Background}_{\ \text{Intracellular Anterior}}$$

$$\text{Posterior Cortical Actin} = \frac{1}{10}\sum_{i=1}^{10} [\text{Posterior Cortical Actin Peak}_{\ i}] - \text{Background}_{\ \text{Intracellular Posterior}}$$

We then calculated the asymmetry of cortical Actin in the posterior pole with the following formula:

$$\text{Cortical Actin Ratio} = \frac{\text{Posterior Cortical Actin}}{\text{Anterior Cortical Actin}}$$

Actin asymmetry was measured at late anaphase when posterior Actin asymmetry had reached its maximum (see **Fig. 1f, g** and Extended Data Fig. 1e-h)

Cortical Actin Asymmetry Quantification in Mud Mutant Analysis ("Linescan" Method)

Maximum projection of *mud* mutant SOP cells expressing Lifeact::mCherry have been selected to measure the cortical peak Actin intensities (**Fig. 2g** and Extended Data Fig. 5f). At anaphase, one linescan of 20 pixels thick has been acquired in the center of the dividing cell.

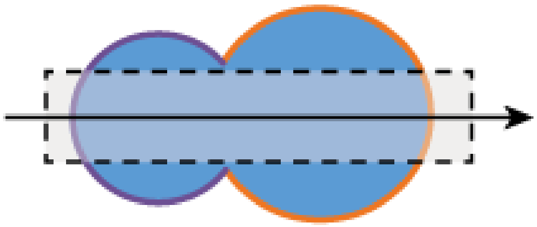

The extracellular background intensities have been determined by taking the mean intensity from a small circle ROI region (≈2.5µm of diameter) outside the cell. For each pole (anterior and posterior), we determined the cortical peak intensities. The cortical peak intensities have been corrected by the intracellular background intensities (determined by taking the mean intensity from small circle ROI regions (≈2.5µm of diameter) inside each anterior and posterior cell pole, respectively).

$$\text{Anterior Cortical Actin Peak} \\ = \text{Anterior Peak} - \text{Background}_{\text{Intracellular Anterior}} - \text{Background}_{\text{Extracellular}}$$

$$\text{Posterior Cortical Actin Peak} \\ = \text{Posterior Peak} - \text{Background}_{\text{Intracellular Posterior}} \\ - \text{Background}_{\text{Extracellular}}$$

Actin asymmetry was computed as the normalized enrichment of Actin in the posterior cortex relative to the anterior according to:

$$\text{Posterior Cortical Actin Asymmetry} = \frac{\text{Posterior Cortical Actin} - \text{Anterior Cortical Actin}}{\text{Posterior Cortical Actin} + \text{Anterior Cortical Actin}}$$

We then calculated the asymmetry of daughter cell size (being larger in the PIIA posterior cell in *wild-type*) with the following formula:

$$\text{Daughter Size Ratio} = \frac{\text{PIIA Projected Area}}{\text{PIIB Projected Area}}$$

For *mud* mutant SOP cells, because the cell can be randomly bisected through the freely rotating spindle, we considered the anterior and the posterior poles as the pole with more Pon signal and the pole with least Pon signal, respectively.

Correlative analysis Daughter Size vs Actin Asymmetry

Maximum projection of late anaphase SOP cells expressing Lifeact::mCherry have been selected to measure the cortical peak Actin intensities. At anaphase, one line scan of 20 pixels thick has

been acquired in the centre of the dividing cell (similar to the "Linescan" Method described above). The extracellular background intensities have been determined by taking the mean intensity from a small circle ROI region (≈2.5µm of diameter) outside the cell. For each pole (anterior and posterior), we determined the cortical peak intensities.

$$\text{Anterior Cortical Actin Peak} = \text{Anterior Peak} - \text{Background}_{\text{Extracellular}}$$
$$\text{Posterior Cortical Actin Peak} = \text{Posterior Peak} - \text{Background}_{\text{Extracellular}}$$

Actin asymmetry was computed as the normalized enrichment of Actin in the posterior cortex relative to the anterior according to:

$$\Delta\text{I} = \text{Cortical Actin Asymmetry} = \frac{\text{Posterior Cortical Actin} - \text{Anterior Cortical Actin}}{\text{Posterior Cortical Actin} + \text{Anterior Cortical Actin}}$$

For correlative measurements of daughter-cell size asymmetry versus Actin asymmetry, and exploration of conditions where size asymmetry is inverted, we rather plotted the normalized size asymmetry Δ, calculated as:

$$\Delta\text{a} = \text{Daughter Size Asymmetry} = \frac{\text{PIIA Projected Area} - \text{PIIB Projected Area}}{\text{PIIA Projected Area} + \text{PIIB Projected Area}}$$

Note that both ΔI and Δa ranges are $-1 \leq \Delta\text{a} \leq 1$ and $-1 \leq \Delta\text{I} \leq 1$.

Linescan Intensity Plots

All linescans in this report has been plotted following the SOP dividing axis (from anterior to posterior) with 20-pixel thick in order to account at maximum the polar cortical proteins.

Cortical Actin Overtime

Maximum projection of SOP cells expressing Lifeact::mCherry with the Neuralized promoter have been selected to measure the cortical Actin dynamics (**Extended Data Fig. 1f**). Cortical Actin enrichment has been measured by means of one linescan (20 pixels thick drawn in the center of the dividing cell) from Metaphase to the end of cytokinesis with a custom-designed ImageJ/FiJi macro. The extracellular background intensities have been determined by taking an average of points to the left of the anterior cortical peak position outside the cell. For each timepoints, the anterior and the posterior cortical peak intensities have been detected in Excel with custom VBA code.

We then calculated the asymmetry of cortical Actin in the posterior pole with the following formula:

$$\text{Posterior Actin Enrichment} = \frac{(\text{Posterior Cortical Actin Peak}) - (\text{Anterior Cortical Actin Peak})}{(\text{Anterior Cortical Actin Peak})} \times 100$$

Cortical Thickness

SOP cells expressing Lifeact::mCherry with the Neuralized promoter have been selected to measure the cortical thickness (Extended Data Fig. 1i, j). At anaphase, for each pole (**Anterior** and **Posterior**), ten linescans (of 5-pixel width each) perpendicular to the cell contour (and excluding the Actin ring) have been acquired on one centered z-plane from the whole z-stacks using a custom-designed ImageJ/FiJi macro.

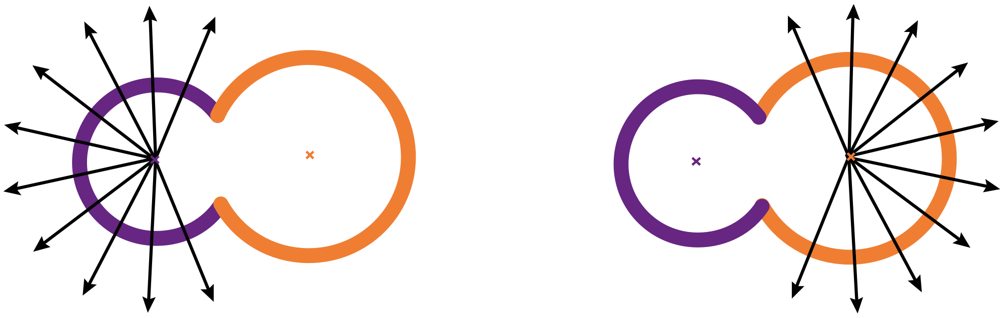

Extracellular background intensities have been determined by taking an average of 5 points to the right of the cortical peak position. After background subtraction, cortical peak positions are detected (usually with highest intensity) and the entire Actin cortex is determined by fitting a one component Gaussian function to the four to six points surrounding and including the cortical peak pixel. Cortical thickness is approximated by the Full Width Half Maximum (FWHM) of the obtained Gaussian function. All the Gaussian fits have a minimum R^2 of 0.999. Note that the Full Width Half Maximum is an over-estimation of the real thickness of the cell cortex, this measurement meant to compare the cortical 'thicknesses' between the two poles and not to provide an exact measure of the Actin cortex width.

Local Curvature vs Cortical Actin Intensity in wild-type

Maximum projection of late anaphase SOP cells expressing Lifeact::mCherry with the Neuralized promoter have been selected to measure the cortical Actin intensities. Cell segmentation and measurement of cortical Actin intensity has been performed using a custom-code written on ImageJ/FiJi using for the segmentation part the Squassh Segmentation plugin (available online) developed in the lab of I. Sbalzarini[1]. In brief, the macro segments overtime the cell contour from a maximum projection of the Lifeact signal, creating a snake around the cell contour, and recording the ROI coordinates. We then measure the cortical Actin Intensity along this segmented contour with a determined thickness (here 10 pixel thick) for each cell poles (**Anterior** and **Posterior**). The thickness has been fixed to 10 pixels to ensure that the entire Actin cortex thickness is measured (maximum 5 pixels). For visualization purposes, the cortical Actin has been smoothed over 5 boundary points and normalized by the total averaged value of cortical Actin of each cell.

Local curvature has been measured by an adapted Matlab script developed in Wolfgang Losert lab[2]. In brief, it selects all the ROI coordinates previously generated and computes the curvatures by fitting a circle with the neighbours (20 neighbours away) in the cell contour: For each point along the cell contour, it calculates the curvature by fitting a circle to that neighbour point and the two points that are 10 points away from it. The curvature is then defined as the reciprocal of the radius of that circle.

Color-coded images for both the curvature and the Actin signal intensity have been generated by a custom-code on ImageJ/FiJi together with a lookup table scale file.

Local Curvature vs Cortical Actin Intensity in optogenetic

To measure the cortical Actin intensity, we first processed maximum projection stacks for background and photobleaching correction. Then we draw a line scan 10 pixels thick perpendicular to the cell contour within the circle ROI illuminated with blue light (3µm circle ≈ 15 pixels diameter). We then performed the line scan measurements to quantify the Lifeact signal in the ROI region throughout the whole registered time by a custom-code written on ImageJ/FiJi. For each timepoints, the peak of the cortical Actin in the line scan has been automatically determined and computed by a VBA macro on Excel.

For the curvature, we manually draw the cell contour in Fiji/ImageJ within the illuminated ROI region. We then used the ImageJ BeanShell code (curvature_radius.bsh code available online by Olivier Burri, BioImaging and Optics Platform at Ecole Polytechnique Fédérale de Lausanne (EPFL)) to determine the local curvatures along this cell contour. We then calculated the average curvature of the ROI region for the several timepoints.

Post-Vertical Orbital Length

SEM images of the same magnification (x100) were analysed to measure the length of the post-vertical orbitals between flies in which the cell size asymmetry has been impaired by targeting WAVE into the anterior pole (*w; UAS - GBP::Pon, UAS - GFP::WAVE / +; Neur - Gal4 / +*) and control flies (white). We quantified the bristle length through a custom-code written on ImageJ/FiJi using a segmented line in order to take into account the curvature of the bristles. In the *w; UAS - GBP::Pon, UAS - GFP::WAVE / +; Neur - Gal4 / +* genotype, we could frequently observe split and forked bristles; in this case, we measured the longest fork end to measure the bristle length.

II. Supplementary Information

I. Surface area distribution during wild-type ACD

During *wild-type* division, we observed that the total surface area increases by approximately 20% (**Fig. 1e**). This is expected when a sphere is split into two spheres with volume conservation and can be explained by simple geometric arguments. Indeed, the surface area of a spherical SOP of radius R_0 is

$$A_{SOP} = A_0 = 4\pi {R_0}^2$$

For a symmetric division, the total surface area after division, when the total volume is conserved, increases by approximately 25%:

$$A_{tot}^{sym} = A_1 + A_2 = \left[\left(\frac{1}{2}\right)^{2/3} + \left(\frac{1}{2}\right)^{2/3}\right] A_0 \approx 1.26 A_0$$

In the case of asymmetry division, with one daughter cell being twice bigger than its sibling, this number decreases slightly:

$$\mathrm{A}_{tot}^{asym} = A_1 + A_2 = \left[\left(\frac{1}{3}\right)^{2/3} + \left(\frac{2}{3}\right)^{2/3}\right] A_0 \approx 1.24 A_0$$

This is consistent with the surface area increase determined from our experimental data (**Fig. 1e**). Consistently, the total surface increase drops further for more pronounced asymmetries, as observed in *Drosophila* Neuroblast divisions[3].
Furthermore, our 3D segmentation data reveals that the posterior surface area increases more than the anterior surface area (**Fig. 1e**). This asymmetry in surface area growth has been indirectly reported in *Drosophila* Neuroblasts, where the bigger daughter cell experiences larger polar elongation during anaphase[3].

II. Asymmetric spinde positionning is not enough to explain size asymmetry in SOPs.

The metaphase plate is not centered, but biased towards the anterior pole (Extended Data Fig. 8a-f). Considering that the Metaphase cell is quasi spherical and that cell volume is conserved across the cell cycle (**Fig. 1c**), the volume of the two spherical caps generated by cleaving the mother at the eccentric position determined by the asymmetric spindle is:

$$V_{Anterior\ Cap} = \pi h^2 (r - \frac{h}{3})$$

$$V_{Posterior\ Cap} = V_{SOP} - \ V_{Anterior\ Cap}$$

with r the radius of the SOP mother, h the distance of the metaphase plate to the tip in the anterior pole and V, the volumes of the SOP or the caps (see Figure below). We calculated that the ratio $V_{Posterior}/V_{Anterior}$ of the two spherical cap volumes if it was merely determined by the eccentric position of the metaphase place (determined by h) is 1.39 ± 0.09 (n=15), while the actual value of $V_{Posterior}/V_{Anterior}$ measured in the SOP daughter cells is much higher (1.88 ± 0.2;

n=24). Therefore, asymmetric positioning of the spindle midzone, is not sufficient by itself to generate the observed unequal daughter cell size.

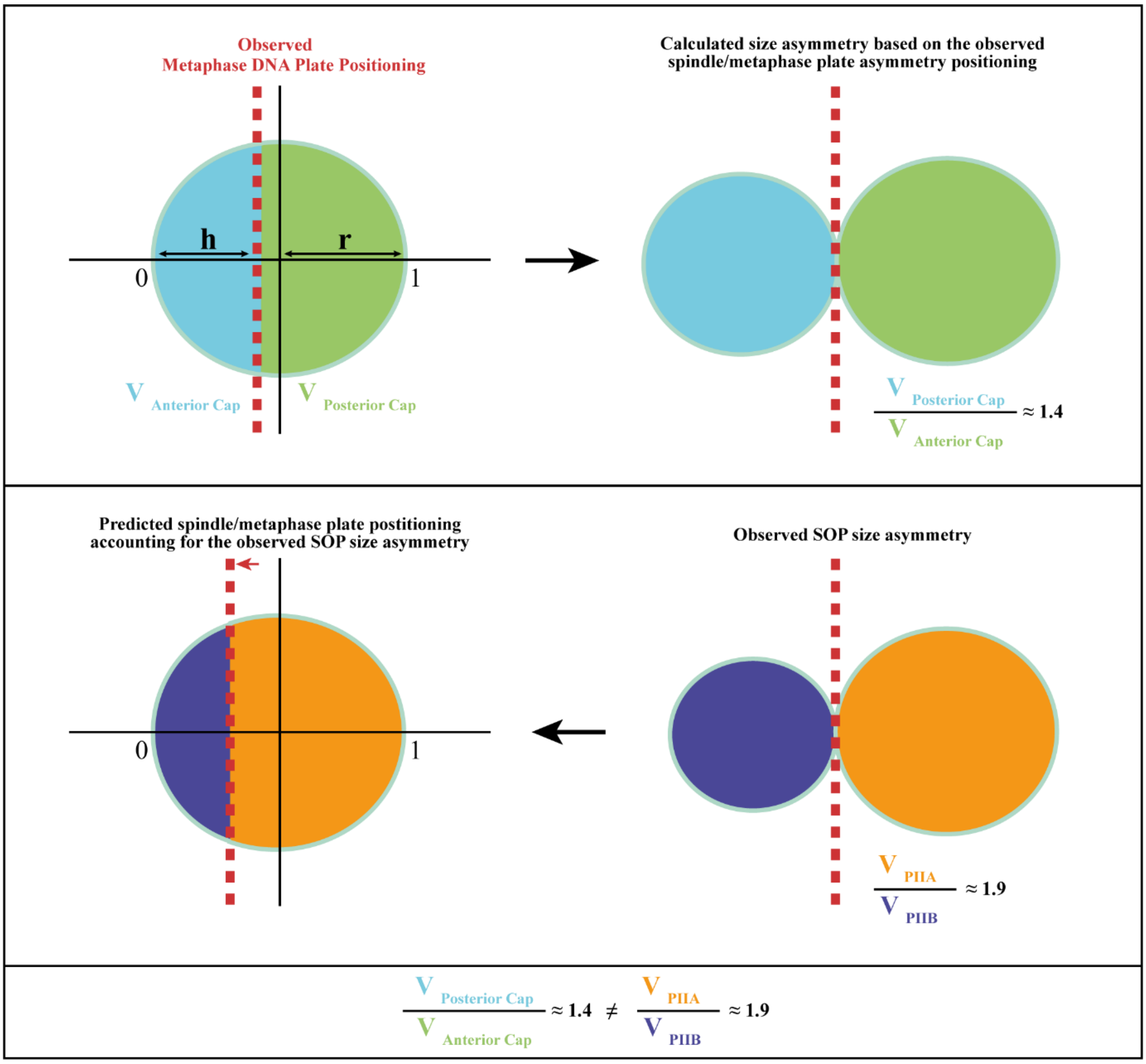

III. Theory

I. Previous Mechanical Models of Cell Division

We first provide a brief overview of previously described models of symmetric eukaryotic cell division that have connected cellular material properties to cell shape and mechanical shape stability. Several previous works have focused on symmetric cell division[4,5] and included the effect of actomyosin flows from the poles into the cytokinetic ring[6]. In these cases, mechanical shape stability was implicitly enforced by fixing the cytokinetic ring at a symmetric position centered between the poles. More recently, a model that combined cortical contractile tension and volume elasticity[7] was introduced and discussed in the context of shape oscillations in symmetrically dividing cells. In this model, polar contraction leads to cytoplasmic flows[8,9] that experience an effective poroelastic resistance. This effect penalizes cell shape asymmetries and thereby stabilizes cell division[7]. Contractile tension and volume elasticity in principle also allow for stable asymmetric cell shapes, if a suitable cortical tension asymmetry is introduced. However, a key prediction of such a model is that the *larger daughter cell* is under a lower contractile surface tension and consequently is expected to have a *lower actomyosin density*, which is inconsistent with our findings in SOP cell divisions: The cortex underneath the *larger pole* presents significantly more Actin (**Fig. 1f, g**), which corresponds to a *larger net actomyosin density* as Myosin densitites are similar at both poles during cytokinesis of SOP cells (**Extended Data Fig. 4c-e**). Recent theoretical work has additionally considered the role of bending rigidity in stabilizing symmetric cell division[10-12]. Therein, it was theoretically shown that symmetric cell division can be stabilized by a finite spontaneous curvature and a sufficiently low surface tension, a result that is compatible with insights from our model.

Theory of Stable Symmetric and Asymmetric Cell Division

In the following, we present a minimal model that connects mechanical cell surface properties to cell shapes during cytokinesis. We show that this model can describe stationary symmetric and asymmetric cell surface geometries and we discuss the mechanical stability of the corresponding force-balanced surface shapes. Starting from experimentally measured Actin asymmetries, the model is then used to estimate material parameter values that quantitatively account for the shape measurements of dividing SOP cells under various experimental conditions.

II.1. Surface geometry and effective energy of the cell surface

Building on previous work[4,7], we consider a simplified surface geometry of two connected spherical caps that represent the two poles of the dividing cell (**Extended Data Fig. 9g**). This geometry is characterized by the radius r_f of the circle connecting the two spherical caps, representing the cleavage furrow radius, as well as by the volumes V_1 and V_2 and surface areas A_1 and A_2 of each spherical cap (**Extended Data Fig. 9g**). Taking into account the volume conservation observed experimentally during SOP cytokinesis (**Fig. 1c**), we consider the family of surface geometries for which the total enclosed volume $V_0 = V_1 + V_2$ is fixed. In this case,

three parameters remain as geometrically independent variables that uniquely specify surface shapes and $A_2(A_1, r_f, V_0)$: the furrow radius r_f, the total volume V_0, and the surface area of one of the caps, which we chose without loss of generality to be A_1. Furthermore, the total volume V_0 defines the characteristic length scale $R_0 = (3V_0/4\pi)^{1/3}$ of the system, which is, in the main text, referred to as the radius R of the SOP cell before division. To connect these surface geometries to the cell surface material properties, we consider an effective shape energy:

$$E(A_1, r_f, V_0) = \sigma_1 A_1 + \sigma_2 A_2 + \frac{\kappa_1}{2} A_1 \left(\frac{2}{R_1} - C_1^{\ 0}\right)^2 + \frac{\kappa_2}{2} A_2 \left(\frac{2}{R_2} - C_2^{\ 0}\right)^2 \\ + 2\pi\sigma_f r_f + pV_0 \tag{1}$$

where the surface material parameters σ_1 and σ_2 denote an effective surface tension, and κ_1 and κ_2 an effective bending rigidity of each cap surface (representing the cell poles of the dividing cell). In general, these material properties can emerge from passive components of the cell surface, such as the cell membrane[13] anchored to the cortex, the degree of Actin branching and cross-linking[14-16], but also from the generation of active tension or moments from the actomyosin cortex[8,17]. However, during division, the effective membrane tension is known to be strongly reduced due to an abundance of folded membrane reservoirs[18-20], such that we consider the forces generated in the actomyosin cortex as the main driver for cell morphogenesis during cell division[21-24]. Passive and active material properties can also contribute to an effective spontaneous curvature $C_1^{(0)}$ and $C_2^{(0)}$, as included in Eq. (1). Spontaneous curvature can generally exist in any thin sheet of material whose intrinsic structure is not up-down mirror symmetric with respect to its midplane[17]. Hence, the broken up-down symmetry of the cellular membrane-cortex architecture in principle provides a sufficient structural background to support a finite spontaneous curvature.

In Eq. (1), we have additionally introduced the line tension along the furrow and the internal pressure excess denoted by σ_f and p, respectively. To determine equilibrium shapes from Eq. (1) in the next section, we take the experimental observations into account that the total volume of SOP cells during cytokinesis is conserved (**Fig. 1c**) and assume that the relaxation of perturbed cell shape asymmetries to stationary configurations is faster than the progression of the furrow constriction. Accordingly, we consider surface variations at constant total volume V_0 and constant furrow radius r_f, while σ_f and p in Eq. (1) represent the corresponding Lagrange multipliers to impose these constraints.

We do not take microscopic processes, such a cross linker dynamics or Actin turnover, explicitly into account. These processes occur on time scales much faster than the overall cell shape changes during cell division and their collective macroscopic effect is captured by the effective material parameters introduced above.

II.2. Stationary cell shapes and shape stability

Using the shape energy $E(A_1, r_f, V_0)$ given in Eq. (1), we now determine stationary surface geometries and discuss how their linear stability under cell surface area perturbations (i.e. pole

size change) depends on the material properties σ_i, κ_i and $C_i^{(0)}$ $(i = 1{,}2)$.

a) General conditions for stationary shapes and linear stability

For the spherical cap geometry, a general minimization of the shape energy $E(A_1, r_f, V_0)$, at constant furrow radius r_f and constant volume V_0, can be performed analytically by using the exact geometric relations:

$$\left.\frac{dR_1}{dA_1}\right|_{r_f, V_0} = 2\pi \cos\theta_1 \frac{R_1^3}{A_1^2} \tag{2}$$

$$\left.\frac{dR_2}{dA_1}\right|_{r_f, V_0} = -\,2\pi \cos\theta_2 \frac{R_2^2 R_1}{A_2^2} \tag{3}$$

$$\left.\frac{dA_2}{dA_1}\right|_{r_f, V_0} = -\frac{R_1}{R_2} \tag{4}$$

$$\left.\frac{d\cos\theta_1}{dA_1}\right|_{r_f, V_0} = \frac{2\pi\, r_f^2}{A_1^2} \tag{5}$$

$$\left.\frac{d\cos\theta_2}{dA_1}\right|_{r_f, V_0} = -\frac{2\pi\, r_f^2 R_1}{A_2^2 R_2} \tag{6}$$

Here, θ_1 and θ_2 denote the opening angles of each spherical cap as depicted in Extended Data Fig. 9g. Equations (2)–(6) hold by symmetry also under an exchange $1 \leftrightarrow 2$ and respect by construction the constraints of constant furrow radius and volume. Accordingly, the Lagrange multipliers σ_f and p Eq. (1) drop out of the following analysis. Equilibrium shapes are given by solutions of a force balance that follows from

$$\left.\frac{dE}{dA_1}\right|_{r_f, V_0} = 0$$

$$\Rightarrow \frac{\sigma_1}{R_1} = \frac{\sigma_2}{R_2}. \tag{7}$$

The force balance Eq. (7) takes the form of a Laplace-pressure balance between the spherical caps with effective tensions σ_i $(i = 1, 2)$ given by

$$\sigma_i = \sigma_i + \frac{\kappa_i}{2R_i^2}\left(2 - R_i C_i^{(0)}\right)\left[2 - R_i C_i^{\;0} \;\; - 8\pi \cos\theta_i \frac{R_i^2}{A_i}\right]. \tag{8}$$

The linear stability of stationary shapes can be determined from

$$\left.\frac{d^2E}{dA_1^2}\right|_{r_f,V_0} = 0, \tag{9}$$

which has to be evaluated for surface geometries that solve the force balance Eq. (7). From Eq. (9), we find that stationary shapes are linearly stable under surface area perturbations if :

$$\begin{aligned} &-\left(\cos\,\theta_1\frac{R_1}{A_1^2}+\cos\theta_2\frac{R_2}{A_2^2}\right)R_1\sigma_2 \\ &-4\pi\left[\kappa_1\frac{R_2r_f^2}{A_1^3}\left(\frac{2}{R_1}-C_1^{\;0}\right)+\kappa_2\frac{R_1r_f^2}{A_2^3}\left(\frac{2}{R_2}-C_2^{\;0}\right)\right] \\ &+4\pi\left[\kappa_1\cos^2\theta_1\frac{C_1^{\;0}\;R_1^2R_2}{A_1^3}+\kappa_2\cos^2\theta_2\frac{C_2^{\;0}\;R_2^2R_1}{A_2^3}\right]>0, \end{aligned} \tag{10}$$

where σ_2 is given in Eq. (8) and we have used Eqs. (1)–(6). Note that for stationary shapes, Eq. (7) implies $R_1\sigma_2 = R_2\sigma_1$, such that the stability criterion Eq. (10) is invariant under an exchange of $1 \leftrightarrow 2$. Consequently, the force balance Eq. (7) and stability criterion Eq. (10) derived here are for arbitrary material parameters independent of choosing the surface area A_1 or A_2 as geometrically independent variable when minimizing the energy Eq. (1).

In the absence of bending rigidity ($\kappa_i = 0$), Eq. (10) leads to a condition for mechanical stability given by $R_1\sigma_2 = R_2\sigma_1 < 0$. This implies that, if solely equipped with an effective tension, a dividing cell would only be mechanically stable if that tension is negative (extensile), i.e. if $\sigma_1 < 0$ and $\sigma_2 < 0$. However, given the extensive experimental[8] and theoretical[25,26] evidences that the actomyosin cortex rather exerts contractile tension ($\sigma_i > 0$), its most likely destabilizing effect must be compensated by additional mechanisms to stabilize cell division (thereby preventing cytokinesis failure where one of the daughter would acquire the whole volume).

b) Symmetric Cell Division: Stability of surfaces with homogeneous material properties

We first discuss the force balance and linear stability Eqs. (7) and (10), respectively, for a symmetrically dividing cell with homogeneous material properties. We establish here that bending rigidity and spontaneous curvature can in general stabilize the cell surface. In particular, we consider a cell with material parameters $\sigma_1 = \sigma_2 = \sigma$, $\kappa_1 = \kappa_2 = \kappa$ and $C_1^{\;0} = C_2^{\;0} = C_0$. From Eqs. (7) and (8), it follows that $R_1 = R_2 = R$, which implies a symmetric surface geometry with $\cos\,\theta_1 = \cos\,\theta_2 = \cos\,\theta$ and $A_1 = A_2 = A$. The condition for mechanical stability of the surface Eq. (10) simplifies in this case to

$$-2\sigma AR^2\cos\theta - \kappa A\cos\theta\;\; 2 - C_0R\;\;^2 - 8\pi\kappa r_f^2\;\; 2 - C_0R\;\; + 16\pi\kappa R^2\cos^2\theta > 0. \tag{11}$$

It follows from Eq. (11) that bending rigidity κ, together with a finite spontaneous curvature $C_0 > 0$, allows for stable symmetric surface shapes. The stability diagram deliniating regions of

shape stability for different fixed furrow radii $r_f = r_f/R_0$ is shown in Extended Data Fig. 9i. For sufficiently large bending rigidities and spontaneous curvature values in a finite interval, symmetric surface geometries are mechanically stable for all furrow radii r_f (blue-shaded region in Extended Data Fig. 9i). For $0 < R_0 C_0 < 2$ only surface geometries, with furrow radii $0 \leq r_f/R_0 < r_f^*$ for some parameter-dependent critical value $r_f^* < 1$, are linearly stable. No stable symmetric geometry at any furrow radius remains for negative spontaneous curvature, $R_0 C_0 < 0$ (gray-shaded region in Extended Data Fig. 9i). On the other hand, moving from the stable blue-shaded region in Extended Data Fig. 9i towards larger values of $R_0 C_0$, only surface geometries with furrow radii $1 \geq r_f/R_0 > r_f^*$ are linearly stable. Symmetric SOP division can be observed when Actin is symmetrically distributed, for example in *lgl*^{3A} condition (**Fig. 1g** and **Fig. 2a-d**). Hence, the stable parameter region found from our minimal model provides a first quantitative indication of the cortical material properties required for stable SOP division.

c) Asymmetric Cell Division: Influence of material properties on shape asymmetry

Evaluating the general condition for mechanical stability from Eq. (10), we also find surfaces with different material properties on either spherical cap that can be mechanically stabilized by bending rigidity and spontaneous curvature. In most cases, the corresponding equilibrium shapes are asymmetric. Qualitatively, these shape asymmetries depend on the surface material properties as follows:

- *Increasing* tension σ_i on a surface *decreases* the size of the corresponding spherical cap, independently of bending rigidity or spontaneous curvature. This is consistent with our Myosin nanobody targeting experiment (Extended Data Fig. 4f-l). Indeed, enriching Myosin in the anterior cortex through GBP-Pon led to a decrease of the anterior pole and thus enhanced the asymmetry of size (Extended Data Fig. 4h,i and 4k,l). Conversely, enriching myosin in the posterior cortex led to more equal-size division (Extended Data Fig. 4j,l).

- The effect of bending rigidity κ_i on size asymmetry depends on the spontaneous curvature $C_i^{\,0}$. In particular, when the spontaneous radius of curvature of a given surface is larger than the cap radius R_i, an *increase* in bending rigidity tends to *increase* the radius of that cap. Conversely, when the spontaneous radius of curvature of a given surface is smaller than the cap radius R_i, an *increase* in bending rigidity tends to *decrease* the radius of that cap.

These aspects are quantitatively illustrated in Extended Data Fig. 9j, where we depict the projected surface area asymmetry $\Delta a = (a_2 - a_1)/(a_1 + a_2)$ (see Extended Data Fig. 9g), while varying an asymmetry in bending rigidity $\Delta\kappa = (\kappa_2 - \kappa_1)/2$ and surface tension $\Delta\sigma = (\sigma_2 - \sigma_1)/2$ between either surface. This corresponds to a variation of the material properties of each surface according to to $\kappa_1 = \kappa_m - \Delta\kappa$, $\kappa_2 = \kappa_m + \Delta\kappa$, $\sigma_1 = \sigma_m - \Delta\sigma$ and $\sigma_2 = \sigma_m + \Delta\sigma$, for fixed mean tension σ_m and mean bending rigidity κ_m. For the spontaneous curvature chosen in this example ($C_1^{(0)} = C_2^{(0)} = 1.8/R_0$), a larger bending rigidity leads to a larger spherical cap sizes, i.e. $\Delta\kappa > 0 \Rightarrow \Delta a > 0$ and $\Delta\kappa < 0 \Rightarrow \Delta a < 0$. Additionally, we see from Extended Data Fig. 9j (black dashed line) that inhomogeneous material properties can also give rise to stable

symmetric surface shapes. In these cases, the tendency of surface tension to reduce spherical cap size is exactly balanced by the tendency of bending rigidity to increase spherical cap size for the chosen spontaneous curvature.

II.3. Comparison with experiments

In this section, we discuss the quantitative comparison between our minimal model and experimental cell size measurements. To this end, we first introduce a parameterization of the material parameters in terms of Actin density. In second step, we describe the fitting procedure and discuss the results.

a) Parameterization of material parameters in the model

To connect the presented minimal model with the Actin-dependent cell shapes observed in experiments, we now introduce a parameterization of the material parameters σ_i, κ_i and C_{i}^{0} in terms of measured Actin densities.

First, we take into account the presence of active contractile tension in the actomyosin cortex[8], as well as the fact that Actin architecture can affect cell surface tension[27]. We therefore consider a linear dependence:

$$\sigma_1 = \alpha_\sigma I_1 \qquad \sigma_2 = \alpha_\sigma I_2, \tag{12}$$

where I_i represents Actin densities measured in terms of fluorescent intensities at the cell poles and $\alpha_\sigma > 0$ denotes a phenomenological tension coefficient that depends on microscopic details. For simplicity, we have chosen this parameter to be the same on both cell surfaces and we have assumed that Actin-independent contributions to tension are small, such that $\sigma_i = 0$ if $I_i = 0$.

The parameterization Eq. (12) is also consistent with our experimental observations. Indeed, during *wild-type* division, we observed asymmetric blebbing phenomena at the end of the cytokinesis with more numerous and larger blebs in the posterior pole (**Extended Data Fig. 1e** and **Extended Data Fig. 9a,b**). It has been previously reported that size of the expanding blebs is a function of the cortical tension[28], therein, blebs have been proposed to be actual reporters of local tension[28-31]. Additionally, we performed cortical laser ablation experiments to rupture the cortex in late anaphase. Consistently, laser-induced blebs in the posterior cortex were measured to be twice as large as laser-induced blebs in the anterior cortex (**Extended Data Fig. 9c,d**). Therefore, larger blebs at the posterior pole of *wild-type* dividing SOP cells suggest that the net cortical tension is larger in the posterior cortex.

Similarly, we consider a scenario in which the bending rigidity is dominated by Actin-dependent contributions and increases linearly with Actin according to

$$\kappa_1 = \alpha_\kappa I_1 \qquad \kappa_2 = \alpha_\kappa I_2, \tag{13}$$

where $\alpha_\kappa > 0$ denotes a phenomenological bending rigidity coefficient.

For the parameterization of the spontaneous curvature ${C_\mathrm{i}}^0$, two aspects are taken into account: (*i*) Mechanical stability restricts ${C_\mathrm{i}}^0$ to a fairly narrow parameter regime (Extended Data Fig. 9i) and (*ii*) active surface theory suggests that the renormalization of spontaneous curvature through active moments is to first order independent of active chemical potential differences[17]. Because the latter can be associated with the local cortical actomyosin concentration[32], we assume the spontaneous curvature is independent of Actin and homogeneous across the whole surface: $C_1^{(0)} = C_2^{(0)} = C_0$. Finally, we note that both parameters α_σ and α_κ will enter the force balance condition linearly when solving for equilibrium shapes (see Eqs. (7) and (8)). Therefore, stationary shapes of this model are only a function of the two dimensionless parameters $R_0 C_0$ and

$$\alpha = \frac{\alpha_\kappa}{\alpha_\sigma R_0^2} = \frac{\kappa_i}{\sigma_i R_0^2}, \tag{14}$$

which are in next step fitted to the experimental data.

b) Fitting procedure and results

Using the spherical cap model with the Actin-parameterization described in the previous section and fixing the furrow radius to $r_f = 0.6R_0$, we evaluated the least squares error of projected area asymmetries that emerge in our minimal model when varying intensity asymmetries $\Delta I = (I_2 - I_1)/(I_1 + I_2)$ (Extended Data Fig. 9k). From the best fit (red dot in Extended Data Fig. 9k), we find $\alpha = 7.1 \pm 0.8$ (fitting parameter defined in Eq. (14)) and $R_0 C_0 = 1.8 \pm 0.1$. The parameter variability $\pm x$ corresponds to standard deviations of the fit parameters that were obtained by repeating the least square minimization 100 times on bootstrapped samples[33] of the cell shape measurement points in Extended Data Fig. 9h. For cell radii $R_0 \approx 5\,\mu$m, the fit result implies a spontaneous curvature of $C_0 \approx 0.36\,\mu\mathrm{m}^{-1}$. From the parameter α an effective cortical bending rigidity κ can be estimated as well. Because the fitted model includes symmetric divisions, where $I_1 = I_2$, we can identify the ratios $\kappa/(\sigma R_0^2) \simeq \alpha_\kappa/(\alpha_\sigma R_0^2) = \alpha$ and estimate the cortical bending rigidity κ using previously measured values of cortical tension ($\sigma \approx 1\,\mathrm{mN/m}$[27,34]). From $\kappa = \alpha\sigma R_0^2$, we then find a cortical bending rigidity of $\kappa \approx 4 \times 10^7 k_B T$. This value is much larger than typical bending rigidities of passive membranes ($\approx 10\,k_B T$), as was also previously noted in [11]. A passive bending rigidity of the thin cortical surface can alternatively be estimated in a thin shell limit as $\kappa_E = Eh^3$, where E denotes the Young compression modulus and h the height of the cell cortex. The Young modulus on the other hand can be estimated from an equilibrium equipartition-scaling argument for semi-flexible polymers as $E = \lambda_p k_B T/(4\pi\ \xi^4) \approx 3 \times 10^5$ Pa, where we have used the persistence length of Actin filaments ($\lambda_p \approx 10\,\mu\mathrm{m}$[35]) and an estimated mesh size $\xi \approx 10\,\mathrm{nm}$. For a cortical height of $h \approx 300\,\mathrm{nm}$[27], we then find $\kappa_E \approx 2 \times 10^6 k_B T$, which is much smaller than the value determined by our fit ($\kappa \approx 4 \times 10^7 k_B T$), suggesting an important role for active moments in modifying the cortical stiffness in response to bending deformations.

II.4. Mechanical stability of surfaces with arbitrary neck radii

As indicated by the stability diagram of symmetric surface geometries, the found fitting parameters (red dot in Extended Data Fig. 9k) are only sufficient to stabilize surface geometries with furrow radii $r_f \lesssim 0.75R_0$. In the context of the present model, three non-exhaustive scenarios can be discussed with regards to the mechanical stability of equilibrium shapes for all neck radii:

1) We have assumed that the equilibration of cell-sizes is faster than the cytokinetic ring closure dynamics, which allowed us to discusse equilibrium shapes at fixed furrow radii. However, a sufficiently fast progression through early cytokinetic ring closure towards surface geometries with stable furrow radii $r_f \lesssim 0.75R_0$ is also conceivable. In this case, daughter-cell surfaces would not have enough time to develop a significant asymmetry and shape instabilities at large furrow radii would be surpressed.

2) Our model additionally allows for a mechanically stable scenario in which an increase in Actin increases bending rigidity, as described in the main text and in Section. III.II.3.a, ($\kappa_1 = \alpha_\kappa I_1$ and $\kappa_2 = \alpha_\kappa I_2$), but tension *decreases* with Actin density according to

$$\sigma_1 = \sigma_0 - \alpha_\sigma I_1 \qquad \sigma_2 = \sigma_0 - \alpha_\sigma I_2, \tag{15}$$

where $\alpha_\sigma > 0$. In this model, the tension σ_0 present in the absence of Actin becomes an additional independent parameter as compared to the scenario discussed in Section. III.II.3.a. Note that in general the regulation of tension and bending rigidity through Actin could be a non-monotonous function and we can interpret any parameterization such as Eqs. (12), (13) or (15) as a linearization of this function around Actin densities that are present during cytokinesis. Interestingly, previous work studying the competition between the two populations of Actin (Formin-mediated Actin and Arp2/3-mediated Actin)[36], changes in the filament length distribution (short, branched Actin filaments)[27], the network architecture[37], as well as cross-linking properties of Arp2/3 Actin networks[38], suggests that branched Actin may lead to a reduction in the effective network tension[39,40], just as reflected by the parameterization Eq. (15).

As described in Section. III.II.2.c, a reduction in spherical cap tension leads in all cases to a cap size increase, such that this scenario is qualitatively compatible with some experimental observations (Extended Data Fig. 4f-l). Furthermore, we find numerically from our model that in the regime of $C_0R_0 \approx 2$ mild tension asymmetries give rise to substantial cap size asymmetries. Hence, for values of spontaneous curvature $C_0R_0 \gtrsim 2$, where surfaces are stable at all furrow radii, but Actin-dependent bending rigidity asymmetries alone are too weak to explain the experimental data, an Actin-dependent reduction in tension as given in Eq. (15) would be sufficient to compensate. To demonstrate this with a concrete example, we use our model with $C_0R_0 = 2.2$, bending rigidity as in Eq. (13) with $\alpha_\kappa/(\sigma_0 R_0^2) = 2.5$, as well as Eq. (15) with $\alpha_\sigma/\sigma_0 = 0.6$. The resulting shape curve as a function of Actin density asymmetries $\Delta I = (I_2 - I_1)/(I_1 + I_2)$ together with experimental data is shown in Extended Data Fig. 9l. Note, due to the off-set tension σ_0 in Eq. (15), different pairs of Actin densities (I_1, I_2)

that yield the same values of normalized density difference $\Delta I = (I_2 - I_1)/(I_1 + I_2)$ can now give rise to different projected area asymmetries $\Delta a = (a_2 - a_1)/(a_1 + a_2)$. The standard deviation of the resulting distribution in Δa is indicated by the error bars in Extended Data Fig. 9l.

3) Finally, we may consider for completeness a scenario in which an increase in Actin density reduces cortical bending rigidity. Here, we take into account that the linear stability diagram Extended Data Fig. 9i indicates spontaneous curvature values $2 \lesssim C_0 R_0 \lesssim 5$ (blue shaded region) robustly provide mechanical stability of surface geometries at all furrow radii. However, as we have discussed in Section. III.II.2.c), spherical cap sizes increase for such rather large spontaneous curvature values only if bending rigidity decreases. Because larger cells are in experiments associated with larger Actin density, this alternative scenario therefore requires that Actin *reduces* local bending rigidity.

This alternative scenario can also describe experimentally observed Actin-dependent cell size asymmetries. To demonstrate this, we choose in the following a spontaneous curvature in the fully stable parameter regime of the stability diagram Extended Data Fig. 9i ($R_0 C_0 = 4$), parameterize the contractile tensions on each spherical cap as before ($\sigma_1 = \alpha_\sigma I_1$ and $\sigma_2 = \alpha_\sigma I_2$), and consider bending rigidities of the form:

$$\kappa_1 = \kappa_0 - \alpha_\kappa I_1 \qquad \kappa_2 = \kappa_0 - \alpha_\kappa I_2 \tag{16}$$

where I_1 and I_2 denote Actin densities on each spherical cap. In Eq. (16), $\alpha_\kappa > 0$ leads to a decrease in bending rigidity around an Actin-independent value κ_0 when the Actin density increases. Three independendent parameters have to be specified in this scenario, which we set here as a concrete example to $R_0 C_0 = 4$, $\kappa_0/(\alpha_\sigma R_0^2) = 2.5$ and $\alpha_\kappa/(\alpha_\sigma R_0^2) = 1$. The resulting shape curve for varying the normalized Actin density difference $\Delta I = (I_2 - I_1)/(I_1 + I_2)$ is shown together with experimental projected area asymmetry measurements in Extended Data Fig. 9m.

Supplementary References

1 Rizk, A. *et al.* Segmentation and quantification of subcellular structures in fluorescence microscopy images using Squassh. *Nat Protoc* **9**, 586-596, doi:10.1038/nprot.2014.037 (2014).

2 Driscoll, M. K. *et al.* Cell shape dynamics: from waves to migration. *PLoS Comput Biol* **8**, e1002392, doi:10.1371/journal.pcbi.1002392 (2012).

3 Connell, M., Cabernard, C., Ricketson, D., Doe, C. Q. & Prehoda, K. E. Asymmetric cortical extension shifts cleavage furrow position in Drosophila neuroblasts. *Mol Biol Cell* **22**, 4220-4226, doi:10.1091/mbc.E11-02-0173 (2011).

4 Yoneda M, D. K. Tension at the Surface of the Dividing Sea-Urchin Egg. *Journal of Experimental Biology* **57**, 575-587 (1972).

5 Zhang, W. & Robinson, D. N. Balance of actively generated contractile and resistive forces controls cytokinesis dynamics. *Proc Natl Acad Sci U S A* **102**, 7186-7191, doi:10.1073/pnas.0502545102 (2005).

6 Turlier, H., Audoly, B., Prost, J. & Joanny, J. F. Furrow constriction in animal cell cytokinesis. *Biophys J* **106**, 114-123, doi:10.1016/j.bpj.2013.11.014 (2014).

7 Sedzinski, J. *et al.* Polar actomyosin contractility destabilizes the position of the cytokinetic furrow. *Nature* **476**, 462-466, doi:10.1038/nature10286 (2011).

8 Salbreux, G., Charras, G. & Paluch, E. Actin cortex mechanics and cellular morphogenesis. *Trends Cell Biol* **22**, 536-545, doi:10.1016/j.tcb.2012.07.001 (2012).

9 Roubinet, C. & Cabernard, C. Control of asymmetric cell division. *Curr Opin Cell Biol* **31**, 84-91, doi:10.1016/j.ceb.2014.09.005 (2014).

10 Almendro-Vedia, V. G., Monroy, F. & Cao, F. J. Mechanics of constriction during cell division: a variational approach. *PLoS One* **8**, e69750, doi:10.1371/journal.pone.0069750 (2013).

11 Almendro-Vedia, V. G., Monroy, F. & Cao, F. J. Analytical results for cell constriction dominated by bending energy. *Phys Rev E Stat Nonlin Soft Matter Phys* **91**, 012713, doi:10.1103/PhysRevE.91.012713 (2015).

12 Beltrán-Heredia, E., Monroy, F. & Cao-García, F. J. Mechanical conditions for stable symmetric cell constriction. *Physical Review E* **100**, 052408, doi:10.1103/PhysRevE.100.052408 (2019).

13 Helfrich, W. Elastic properties of lipid bilayers: theory and possible experiments. *Z Naturforsch C* **28**, 693-703 (1973).

14 Pujol, T., du Roure, O., Fermigier, M. & Heuvingh, J. Impact of branching on the elasticity of actin networks. *Proc Natl Acad Sci U S A* **109**, 10364-10369, doi:10.1073/pnas.1121238109 (2012).

15 Razbin, M., Falcke, M., Benetatos, P. & Zippelius, A. Mechanical properties of branched actin filaments. *Phys Biol* **12**, 046007, doi:10.1088/1478-3975/12/4/046007 (2015).

16 Gardel, M. L. *et al.* Elastic behavior of cross-linked and bundled actin networks. *Science* **304**, 1301-1305, doi:10.1126/science.1095087 (2004).

17 Salbreux, G. & Jülicher, F. Mechanics of active surfaces. *Physical Review E* **96**, 032404, doi:10.1103/PhysRevE.96.032404 (2017).

18 Clark, A. G., Dierkes, K. & Paluch, E. K. Monitoring actin cortex thickness in live cells. *Biophys J* **105**, 570-580, doi:10.1016/j.bpj.2013.05.057 (2013).

19 Boucrot, E. & Kirchhausen, T. Endosomal recycling controls plasma membrane area during mitosis. *Proc Natl Acad Sci U S A* **104**, 7939-7944, doi:10.1073/pnas.0702511104 (2007).

20 Taubenberger, A. V., Baum, B. & Matthews, H. K. The Mechanics of Mitotic Cell Rounding. *Frontiers in Cell and Developmental Biology* **8**, doi:10.3389/fcell.2020.00687 (2020).

21 Mohan, K., Iglesias, P. A. & Robinson, D. N. Separation anxiety: stress, tension and cytokinesis. *Exp Cell Res* **318**, 1428-1434, doi:10.1016/j.yexcr.2012.03.028 (2012).

22 Chugh, P. & Paluch, E. K. The actin cortex at a glance. *J Cell Sci* **131**, doi:10.1242/jcs.186254 (2018).

23 Pollard, T. D. & Cooper, J. A. Actin, a central player in cell shape and movement. *Science* **326**, 1208-1212, doi:10.1126/science.1175862 (2009).
24 Haase, K. & Pelling, A. E. The role of the actin cortex in maintaining cell shape. *Commun Integr Biol* **6**, e26714, doi:10.4161/cib.26714 (2013).
25 Dasanayake, N. L., Michalski, P. J. & Carlsson, A. E. General mechanism of actomyosin contractility. *Phys Rev Lett* **107**, 118101, doi:10.1103/PhysRevLett.107.118101 (2011).
26 Thoresen, T., Lenz, M. & Gardel, M. L. Thick filament length and isoform composition determine self-organized contractile units in actomyosin bundles. *Biophys J* **104**, 655-665, doi:10.1016/j.bpj.2012.12.042 (2013).
27 Chugh, P. *et al.* Actin cortex architecture regulates cell surface tension. *Nat Cell Biol*, doi:10.1038/ncb3525 (2017).
28 Tinevez, J. Y. *et al.* Role of cortical tension in bleb growth. *Proc Natl Acad Sci U S A* **106**, 18581-18586, doi:10.1073/pnas.0903353106 (2009).
29 Jean-Yves Tineveza, b., 1, Ulrike Schulzea,b,1,2, Guillaume Salbreuxc,1,3, Julia Roenscha,b, Jean-François Joannyc,4, & Palucha, a. E. Role of cortical tension in bleb growth. *PNAS* (2009).
30 Charras, G. T., Coughlin, M., Mitchison, T. J. & Mahadevan, L. Life and times of a cellular bleb. *Biophys J* **94**, 1836-1853, doi:10.1529/biophysj.107.113605 (2008).
31 Peukes, J. & Betz, T. Direct measurement of the cortical tension during the growth of membrane blebs. *Biophys J* **107**, 1810-1820, doi:10.1016/j.bpj.2014.07.076 (2014).
32 Saha, A. *et al.* Determining Physical Properties of the Cell Cortex. *Biophys J* **110**, 1421-1429, doi:10.1016/j.bpj.2016.02.013 (2016).
33 Efron, B. Bootstrap Methods: Another Look at the Jackknife. *The Annals of Statistics* **7**, 1-26, 26 (1979).
34 Fischer-Friedrich, E. *et al.* Rheology of the Active Cell Cortex in Mitosis. *Biophys J* **111**, 589-600, doi:10.1016/j.bpj.2016.06.008 (2016).
35 Ott, A., Magnasco, M., Simon, A. & Libchaber, A. Measurement of the persistence length of polymerized actin using fluorescence microscopy. *Physical Review E* **48**, R1642-R1645, doi:10.1103/PhysRevE.48.R1642 (1993).
36 Carlier, M. F. & Shekhar, S. Global treadmilling coordinates actin turnover and controls the size of actin networks. *Nat Rev Mol Cell Biol* **18**, 389-401, doi:10.1038/nrm.2016.172 (2017).
37 Reymann, A. C. *et al.* Actin Network Architecture Can Determine Myosin Motor Activity. *Science* **336**, 1310-1314, doi:10.1126/science.1221708 (2012).
38 Bendix, P. M. *et al.* A quantitative analysis of contractility in active cytoskeletal protein networks. *Biophys J* **94**, 3126-3136, doi:10.1529/biophysj.107.117960 (2008).
39 Chaigne, A. *et al.* A soft cortex is essential for asymmetric spindle positioning in mouse oocytes. *Nat Cell Biol* **15**, 958-966, doi:10.1038/ncb2799 (2013).
40 Cartagena-Rivera, A. X., Logue, J. S., Waterman, C. M. & Chadwick, R. S. Actomyosin Cortical Mechanical Properties in Nonadherent Cells Determined by Atomic Force Microscopy. *Biophys J* **110**, 2528-2539, doi:10.1016/j.bpj.2016.04.034 (2016).
