# Supplementary material for: Polarized branched Actin modulates cortical mechanics to produce unequal-size daughters during asymmetric division
Source: Nat Cell Biol. 2023 Feb 6;25(2):235–45. doi: 10.1038/s41556-022-01058-9 (PMC9928585; doi:10.1038/s41556-022-01058-9)
Supplement: Supplementary file 2 — Reporting Summary [file 41556_2022_1058_MOESM2_ESM.pdf]

nature portfolio

Corresponding author(s): Marcos Gonzalez-Gaitan

Last updated by author(s): Jul 7, 2022

Reporting Summary

Nature Portfolio wishes to improve the reproducibility of the work that we publish. This form provides structure for consistency and transparency in reporting. For further information on Nature Portfolio policies, see our Editorial Policies and the Editorial Policy Checklist.

Statistics

For all statistical analyses, confirm that the following items are present in the figure legend, table legend, main text, or Methods section.

n/a	Confirmed	
☐	☒	The exact sample size (*n*) for each experimental group/condition, given as a discrete number and unit of measurement
☐	☒	A statement on whether measurements were taken from distinct samples or whether the same sample was measured repeatedly
☐	☒	The statistical test(s) used AND whether they are one- or two-sided<br>*Only common tests should be described solely by name; describe more complex techniques in the Methods section.*
☒	☐	A description of all covariates tested
☐	☒	A description of any assumptions or corrections, such as tests of normality and adjustment for multiple comparisons
☐	☒	A full description of the statistical parameters including central tendency (e.g. means) or other basic estimates (e.g. regression coefficient) AND variation (e.g. standard deviation) or associated estimates of uncertainty (e.g. confidence intervals)
☐	☒	For null hypothesis testing, the test statistic (e.g. *F*, *t*, *r*) with confidence intervals, effect sizes, degrees of freedom and *P* value noted<br>*Give P values as exact values whenever suitable.*
☒	☐	For Bayesian analysis, information on the choice of priors and Markov chain Monte Carlo settings
☒	☐	For hierarchical and complex designs, identification of the appropriate level for tests and full reporting of outcomes
☐	☒	Estimates of effect sizes (e.g. Cohen's *d*, Pearson's *r*), indicating how they were calculated

Our web collection on statistics for biologists contains articles on many of the points above.

Software and code

Policy information about availability of computer code

Data collection	Software built in the microscope for image acquisition: Slidebook 6 (3i)
Data analysis	ImageJ/FiJi (2.1.0/1.53q), Excel MS Office 2012, MATLAB 2019b (The MathWorks), SigmaPlot 12.5. Figures were generated using Illustrator (Adobe 2021) and videos were generated using Adobe Premiere Pro 2021.<br>Custom codes written in ImageJ and Matlab are available from the corresponding author upon reasonable request. Local curvature has been measured by an adapted Matlab script from Wolfgang Losert lab published in Driscoll et al.

For manuscripts utilizing custom algorithms or software that are central to the research but not yet described in published literature, software must be made available to editors and reviewers. We strongly encourage code deposition in a community repository (e.g. GitHub). See the Nature Portfolio guidelines for submitting code & software for further information.

Data

Policy information about availability of data

All manuscripts must include a data availability statement. This statement should provide the following information, where applicable:

- Accession codes, unique identifiers, or web links for publicly available datasets
- A description of any restrictions on data availability
- For clinical datasets or third party data, please ensure that the statement adheres to our policy

The data that support the findings of this study are available within the paper and its Supplementary Information files. Source data are provided with this study.
Links of publicly available datasets used in this work:
https://flybase.org/

Field-specific reporting

Please select the one below that is the best fit for your research. If you are not sure, read the appropriate sections before making your selection.

☒ Life sciences ☐ Behavioural & social sciences ☐ Ecological, evolutionary & environmental sciences

For a reference copy of the document with all sections, see nature.com/documents/nr-reporting-summary-flat.pdf

Life sciences study design

All studies must disclose on these points even when the disclosure is negative.

Sample size	No sample size pre-calculations were included in our analysis. Sample sizes are based on positive and/or negative controls where statistically significant differences were detected. The sample size has been chosen based on work of other groups using Drosophila Sensory Organ Precursors as a model system.
Data exclusions	As disclosed in the Methods, SOP cells showing spindle orientation defects were excluded from the WAVE nanobody experiment and, for the dshRNAi experiment, only cells with polarity defects were selected (low phenotype penetrance).
Replication	All experiments including data replicates. Micrographs are representative from a set of at least 2 independent experimental rounds performed on different days and were in all cases reproducible.
Randomization	Flies of the same genetic background were kept separate and experiments was carried out on notum obtained from randomly chosen Drosophila pupae of the appropriate genetic background and developmental stage. Our study does not explore the impact of different treatments on subjects, nor did it require sampling individuals that belong to different groups from large populations. As such randomization is not strictly relevant to our analysis.
Blinding	No blinded studies were performed. Blinding is not relevant to this study. The experiments require the investigators to group the data between control and testing condition to quantify differences.

Reporting for specific materials, systems and methods

We require information from authors about some types of materials, experimental systems and methods used in many studies. Here, indicate whether each material, system or method listed is relevant to your study. If you are not sure if a list item applies to your research, read the appropriate section before selecting a response.

Materials & experimental systems

n/a	Involved in the study
☒	☐ Antibodies
☒	☐ Eukaryotic cell lines
☒	☐ Palaeontology and archaeology
☐	☒ Animals and other organisms
☒	☐ Human research participants
☒	☐ Clinical data
☒	☐ Dual use research of concern

Methods

n/a	Involved in the study
☒	☐ ChIP-seq
☒	☐ Flow cytometry
☒	☐ MRI-based neuroimaging

Animals and other organisms

Policy information about studies involving animals; ARRIVE guidelines recommended for reporting animal research

Laboratory animals	Males and Females from Drosophila melanogaster were used for the analysis in our work (Genotypes: see Supplemental Information). All the experiments in sensory organ precursors have been performed on notum during the pupal stage. Experiments on neuroblasts have been performed on L3 larvae. The genotypes used are detailed in Supplementary Information.
Wild animals	This study did not involve wild animals.
Field-collected samples	This study did not involve samples collected from the field.
Ethics oversight	This study did not require Ethics and Animal protection directives.

Note that full information on the approval of the study protocol must also be provided in the manuscript.
